# Stage- and sex-specific transcriptome analyses reveal distinctive sensory gene expression patterns in a butterfly

**DOI:** 10.1186/s12864-021-07819-4

**Published:** 2021-08-02

**Authors:** David A. Ernst, Erica L. Westerman

**Affiliations:** Department of Biological Sciences, University of Arkansas, 72701 Fayetteville, AR USA

**Keywords:** Vision, Phototransduction, Chemoreception, Lepidoptera, Caterpillar, Wing patterning

## Abstract

**Background:**

Animal behavior is largely driven by the information that animals are able to extract and process from their environment. However, the function and organization of sensory systems often change throughout ontogeny, particularly in animals that undergo indirect development. As an initial step toward investigating these ontogenetic changes at the molecular level, we characterized the sensory gene repertoire and examined the expression profiles of genes linked to vision and chemosensation in two life stages of an insect that goes through metamorphosis, the butterfly *Bicyclus anynana*.

**Results:**

Using RNA-seq, we compared gene expression in the heads of late fifth instar larvae and newly eclosed adults that were reared under identical conditions. Over 50 % of all expressed genes were differentially expressed between the two developmental stages, with 4,036 genes upregulated in larval heads and 4,348 genes upregulated in adult heads. In larvae, upregulated vision-related genes were biased toward those involved with eye development, while phototransduction genes dominated the vision genes that were upregulated in adults. Moreover, the majority of the chemosensory genes we identified in the *B. anynana* genome were differentially expressed between larvae and adults, several of which share homology with genes linked to pheromone detection, host plant recognition, and foraging in other species of Lepidoptera.

**Conclusions:**

These results revealed promising candidates for furthering our understanding of sensory processing and behavior in the disparate developmental stages of butterflies and other animals that undergo metamorphosis.

**Supplementary Information:**

The online version contains supplementary material available at 10.1186/s12864-021-07819-4.

## Background

The environment is teeming with information, and the ability to perceive and process this information is critical in shaping the behavior of all animals. Of the various sensory modalities, vision and chemoreception play integral roles in survival and reproduction, including the detection of food sources [1], predator avoidance [2], and locating potential mates [3]. Moreover, both senses are known to drive assortative mating and speciation processes. Visual cues, such as ornaments [4], coloration [5], and courtship displays [6], influence mate choice behaviors and sexual selection in a diverse range of species. Similarly, chemical signals, such as pheromones and cuticular hydrocarbons, have been found to be involved with prezygotic isolation in a wide variety of taxa, ranging from insects [7–9] and annelids [10] to mammals [11–13].

Despite the significant roles that visual and chemical cues play in animal behavior and sexual selection, considerable morphological differences often exist for the sensory structures that detect these cues throughout ontogeny. This is particularly apparent in animals that undergo metamorphosis from larva to adult life stages, such as holometabolous insects [14], crustaceans [15], and many fishes [16]. For instance, in butterflies, the visual organs of the larval stage typically consist of up to six stemmata per eye, each with a lens and seven photoreceptors that form a tiered rhabdom [17], compared to the much more complex adult compound eyes, which consist of hundreds of tightly packed ommatidia, each containing a facet lens and rhabdom composed of nine photoreceptors [18]. These differences are likely in part due to the different ecological niches that each stage fills; larvae typically reside and forage on host plants until pupation, while adults adopt an aerial lifestyle and are mainly focused on finding a mate and reproducing [14].

Although differences in sensory organ morphology and the behavior of animals that undergo metamorphosis are often readily apparent throughout ontogeny, we still have much to learn about the functional and organizational differences in the sensory systems of pre- and post-metamorphosis life stages, especially at the molecular level. Perhaps one of the most promising taxa in which to dissect these differences is the exceptionally diverse Insecta, which is estimated to consist of 5.5 million species [19]. Indeed, much of what we know about the molecular mechanisms underlying vision and chemosensation has been derived from work on insects, including the common fruit fly, *Drosophila melanogaster* (see [20] and [21] for review).

Phototransduction in insects is accomplished in the eye through absorption of light by a visual pigment (rhodopsin), which triggers an enzymatic cascade that ultimately leads to depolarization of photoreceptor cells [20]. The perception of different wavelengths of light is dependent upon opsin structure, with peak sensitivities spanning the visible light spectrum and beyond [22]. By contrast, chemosensation in insects occurs at the olfactory sensilla (the sensory structures involved with smell) typically found on head structures, such as the maxillary palps and antennae, and the gustatory sensilla (the sensory structures involved with taste), which are found throughout the insect body, including on the mouthparts, wings, and legs [23, 24]. Odorants are bound by odorant binding proteins (OBPs) or chemosensory proteins (CSPs) and transported through the sensillar lymph to membrane-bound receptors located on the dendrites of olfactory sensory neurons (OSNs) or gustatory sensory neurons (GSNs) [25, 26].

There are three main types of chemoreceptors on chemosensory neurons that are involved with the detection of chemical stimuli from the external environment in insects: odorant receptors (ORs), gustatory receptors (GRs), and ionotropic receptors (IRs). ORs are the foundation of olfaction and are known to selectively detect a diversity of volatile compounds [27–29]. In combination with a co-receptor (*Orco*) and sensory neuron membrane proteins (SNMPs), some ORs have also been found to be involved with the detection of sex pheromones [30, 31]. Insect GRs belong to the same superfamily as ORs [32] but are primarily involved with tasting bitter compounds [33–35], sugars [36–38], and CO_2_ [39, 40]. Finally, IRs, which are primitive chemoreceptor proteins that evolved from ionotropic glutamate receptors (iGluRs), are known to be involved with both olfaction and gustation, primarily sensing amines, acids, salt, and pheromones [24, 41–43].

Recent work in adult Lepidoptera has focused on elucidating the underpinnings of phototransduction [44] and chemoreception [24, 31, 45, 46], providing a foundation for investigating how sensory systems vary throughout development in an insect order known for its metamorphosis. The squinting bush brown butterfly, *Bicyclus anynana*, is an ideal model to address this issue, as it has rapidly become a fruitful model system for studying development, evolution, and phenotypic plasticity [47–50]. Of particular interest, these butterflies rely heavily on visual and chemical cues for mate choice; mates are selected based on the quality of ultraviolet-reflective eyespot pupils and male-specific pheromones [4, 51, 52]. In addition, previous work has identified differences in the visual systems of male and female adults of different seasonal phenotypes, including differences in eye size, facet lens area, facet number per eye, and opsin and eye development gene expression [53, 54]. Importantly, numerous molecular resources are available for this species, including expressed sequence tags [55], transcriptomes [54], and a reference genome assembly [56], making *B. anynana* amenable to genetic and genomic studies.

**Fig. 1:**
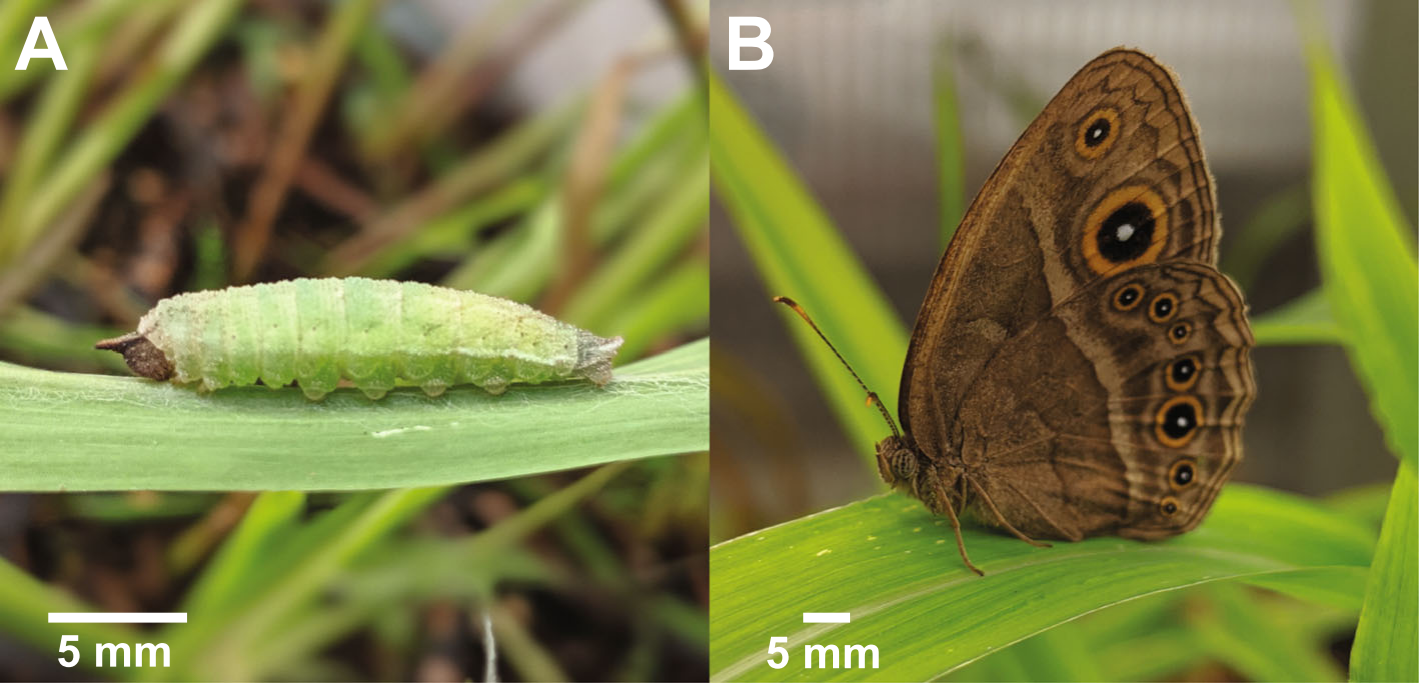
Developmental stages of *B. anynana*. (**A**) Late fifth instar larva. (**B**) Newly eclosed adult. Scale bars are approximate

Here, we characterized the sensory gene repertoire in *B. anynana*, comprising genes known to be linked to vision and chemosensation. Specifically, we first identified vision genes in the *B. anynana* genome involved with phototransduction, eye pigmentation, and eye development, as well as six distinct families of chemosensory genes, consisting of OBPs, CSPs, ORs, IRs, GRs, and SNMPs. We also identified developmental genes that have possibly been co-opted to function as sensory or neural processing genes, such as those known to be involved with wing patterning in *B. anynana* and other butterflies, which are hypothesized to potentially drive speciation and assortative mating by linking wing pattern traits to preference for those traits [57–59]. We then compared the expression patterns of these sensory and developmental genes in the heads of two life stages: late fifth instar larvae and newly eclosed adult butterflies (Fig. 1).

We predicted that genes directly involved with visual processes (e.g., phototransduction) would be upregulated in the adult phenotype, given the much greater level of complexity of adult compound eyes compared to the relative simplicity of larval stemmata. In addition, we hypothesized that larvae might not express the ultraviolet-sensitive opsin that is critical to eyespot quality evaluation during mate choice in adults. For chemosensory genes, we predicted that genes involved with pheromone and fruit detection would be upregulated in adults, as this stage participates in numerous reproductive behaviors and must locate a food source (i.e., ripe or rotting fruit) separate from the host plant. By contrast, we hypothesized that chemosensory genes linked to host plant recognition and foraging behavior would be upregulated in larvae, given that feeding is the dominant behavior during this stage of development. Furthermore, if genes associated with wing patterning in butterflies are also important for behavioral aspects of assortative mating, we predicted that they would be upregulated in the brains of adults. Finally, we aimed to identify candidate visual and chemosensory genes in the adult and larval phenotypes for future investigation into the sensory ecology of these disparate life stages.

## Results

Sequencing generated over 387 million high quality single-end (SE) reads (Additional file 1: Table S1). Adapter trimming removed 87,301 reads (0.02 % of the raw sequenced reads) prior to downstream analysis. Approximately 340 million (88 %) of the remaining trimmed reads mapped to the *B. anynana* genome (v1.2; [56]; http://ensembl.lepbase.org/index.html). Of the 22,642 predicted protein-coding genes in the reference genome, 15,735 (70 %) were overlapped by at least 10 reads across all libraries and used as the expression set for downstream analyses. This gene set corresponded to approximately 12.2 ± 2.3 SD million reads per sample that were used for differential expression analysis (Table S1). Principal components analysis revealed that developmental stage explained 75 % of the variation observed (Additional file 1: Fig. S1). Blast2GO analysis resulted in the functional annotation of 13,498 (60 %) genes in the *B. anynana* genome, with a total of 40,857 gene ontology (GO) terms assigned to genes in the assembly.

### Adult vs. larval heads

**Fig. 2:**
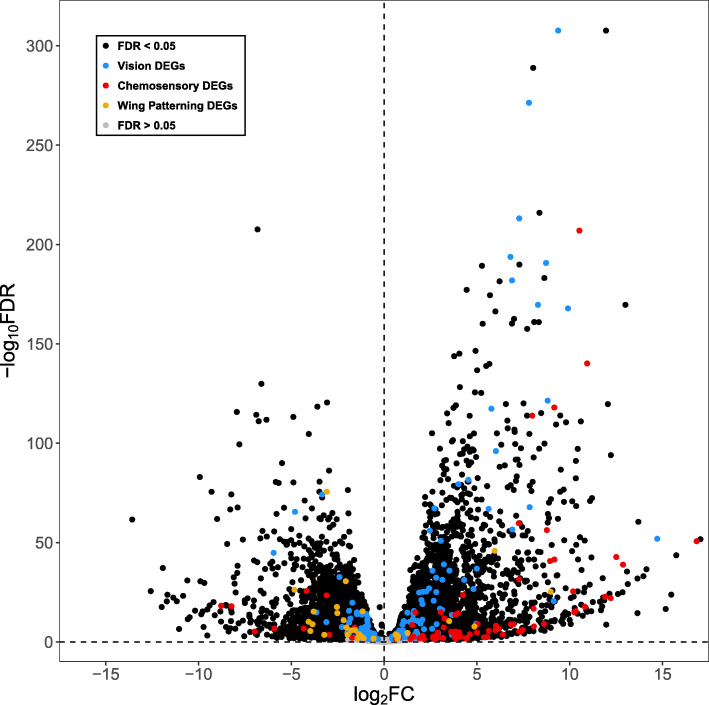
Volcano plot of the false discovery rate (-log_10_FDR) and expression ratio (log_2_FC) for each gene in *B. anynana* adult heads relative to larval heads. Differentially expressed vision, chemosensory, and wing patterning gene homologs are highlighted in blue, red, and orange, respectively. Positive log_2_FC values indicate higher expression in adults, while negative log_2_FC values indicate higher expression in larvae. DEGs = Differentially Expressed Genes. This plot was created with ggplot2 v3.3.2 [60] in R v3.6.2 [61]

A total of 8,384 (53 % of genes in the expression set) genes were differentially expressed between larva and adult stage heads, with 4,348 upregulated in adult heads and 4,036 upregulated in larva heads (FDR < 0.05; Fig. 2; Additional file 2: Table S2). GO enrichment analyses of these upregulated genes found that 255 GO terms were enriched in the heads of adults (Additional file 2: Table S3). When reduced to the most specific terms (i.e., parent functions with a significant child GO term were removed to reduce redundancy), 63 enriched GO terms remained, with the top three terms being oxidation-reduction process (FDR = 1.53 × 10^− 18^), proton transmembrane transporter activity (FDR = 4.12 × 10^− 10^), and potassium ion transmembrane transport (FDR = 1.26 × 10^− 9^) (Table 1; see Additional file 2: Table S4 and Additional file 1: Figs. S2-S4 for full results). By contrast, 212 GO terms were enriched in the heads of larvae (Additional file 2: Table S5). A total of 49 GO terms remained after reduction, with the top three terms being nucleolus (FDR = 4.15 × 10^− 11^), mRNA splicing, via spliceosome (FDR = 4.26 × 10^− 11^), and protein folding (FDR = 1.17 × 10^− 10^) (Table 2; see Additional file 2: Table S6 and Additional file 1: Figs. S5-S7 for full results).

**Table 1:** Top 10 most specific GO terms enriched in adult heads. GO = Gene Ontology, BP = Biological Process, MF = Molecular Function, FDR = False Discovery Rate

GO ID	GO Name	GO Category	FDR
GO:0055114	oxidation-reduction process	BP	1.53 × 10^− 18^
GO:0015078	proton transmembrane transporter activity	MF	4.12 × 10^− 10^
GO:0071805	potassium ion transmembrane transport	BP	1.26 × 10^− 9^
GO:0005506	iron ion binding	MF	1.26 × 10^− 9^
GO:0020037	heme binding	MF	4.01 × 10^− 9^
GO:0005549	odorant binding	MF	5.83 × 10^− 8^
GO:0016705	oxidoreductase activity, acting on paired donors, with incorporation or reduction of molecular oxygen	MF	1.54 × 10^− 7^
GO:0042302	structural constituent of cuticle	MF	5.50 × 10^− 7^
GO:0004930	G protein-coupled receptor activity	MF	5.65 × 10^− 7^
GO:0005249	voltage-gated potassium channel activity	MF	5.05 × 10^− 6^

**Table 2:** Top 10 most specific GO terms enriched in larval heads. GO = Gene Ontology, BP = Biological Process, MF = Molecular Function, CC = Cellular Component, FDR = False Discovery Rate

GO ID	GO Name	GO Category	FDR
GO:0005730	nucleolus	CC	4.15 × 10^− 11^
GO:0000398	mRNA splicing, via spliceosome	BP	4.26 × 10^− 11^
GO:0006457	protein folding	BP	1.17 × 10^− 10^
GO:0003735	structural constituent of ribosome	MF	1.17 × 10^− 10^
GO:0005840	ribosome	CC	2.23 × 10^− 9^
GO:0006364	rRNA processing	BP	6.27 × 10^− 9^
GO:0007275	multicellular organism development	BP	3.24 × 10^− 8^
GO:0003743	translation initiation factor activity	MF	5.98 × 10^− 8^
GO:0005681	spliceosomal complex	CC	1.57 × 10^− 7^
GO:0051082	unfolded protein binding	MF	4.09 × 10^− 7^

### Sex-specific differences within each stage

**Fig. 3:**
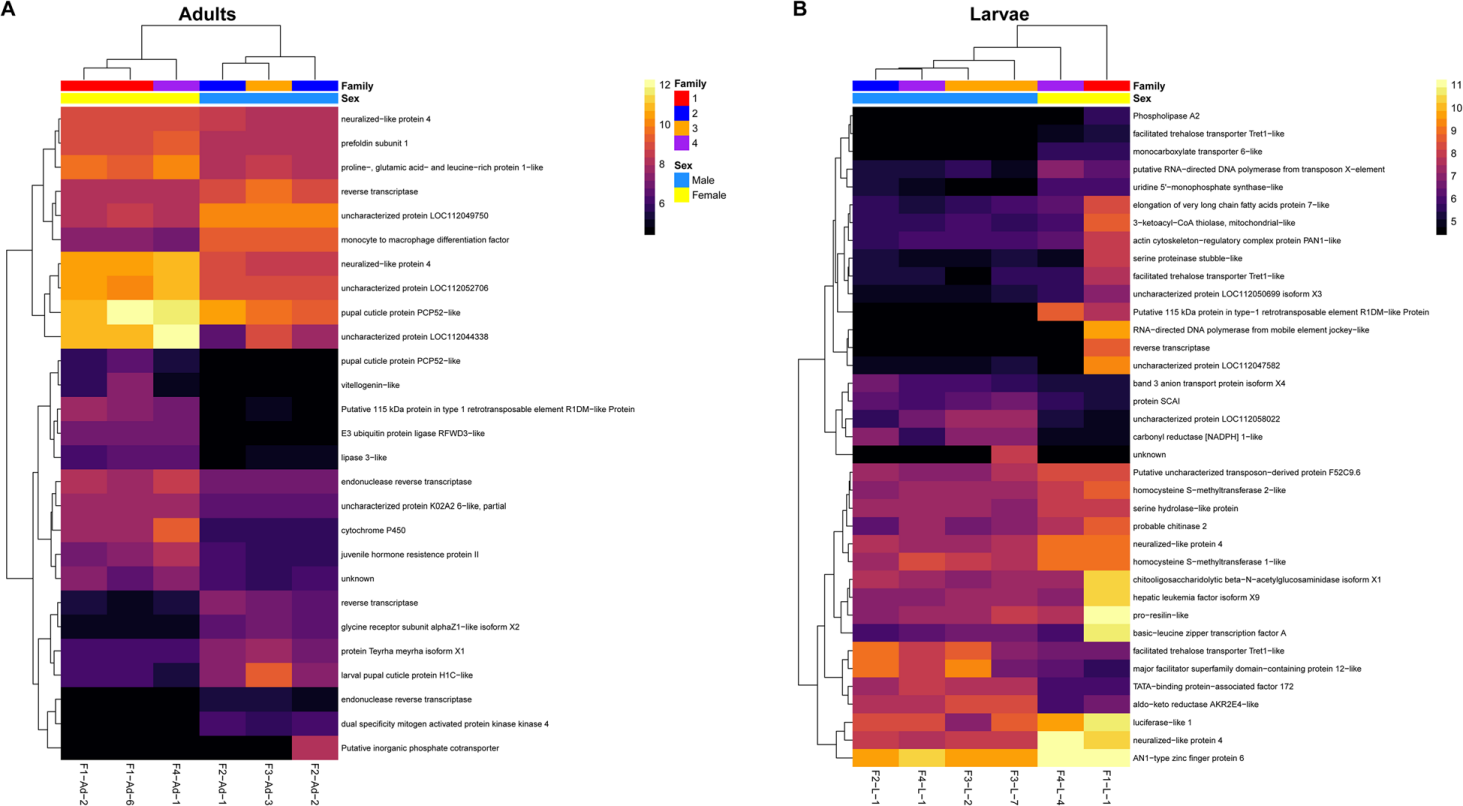
Expression heatmaps of differentially expressed genes for sex-specific comparisons within each developmental stage. (**A**) Male adults vs. female adults. (**B**) Male larvae vs. female larvae. Counts were normalized by variance stabilizing transformation, with warmer colors indicating higher expression. Rows denote individual genes, and columns denote samples, both of which are clustered by gene expression. Family indicates the family from which the sample was derived, and Sex indicates the sex of the sample. Heatmaps were created with pheatmap v1.0.12 [62] in R v3.6.2 [61]

A comparison between male and female adult heads revealed 27 differentially expressed genes, 10 of which were upregulated in male adults, while 17 were upregulated in female adults (FDR < 0.05; Fig. 3A; Additional file 2: Table S7). By contrast, 37 genes were differentially expressed between male and female larval heads, with nine upregulated in male larvae and 28 upregulated in female larvae (FDR < 0.05; Fig. 3B; Additional file 2: Table S8). GO enrichment analyses found no significantly enriched GO terms for the adult or larva differentially expressed gene sets. Furthermore, none of the vision, chemosensory, or wing patterning/development genes identified in the reference genome (see below) were differentially expressed between the sexes of larvae or adults. There were three genes in common between the two stage-specific differentially expressed gene sets, including Putative 115 kDa protein in type-1 retrotransposable element R1DM-like protein (*BANY.1.2.g05985*) and two copies of neuralized-like protein 4 (*BANY.1.2.g11289* and *BANY.1.2.g11290*), all of which were upregulated in female larvae and adults.

### Vision genes

**Fig. 4:**
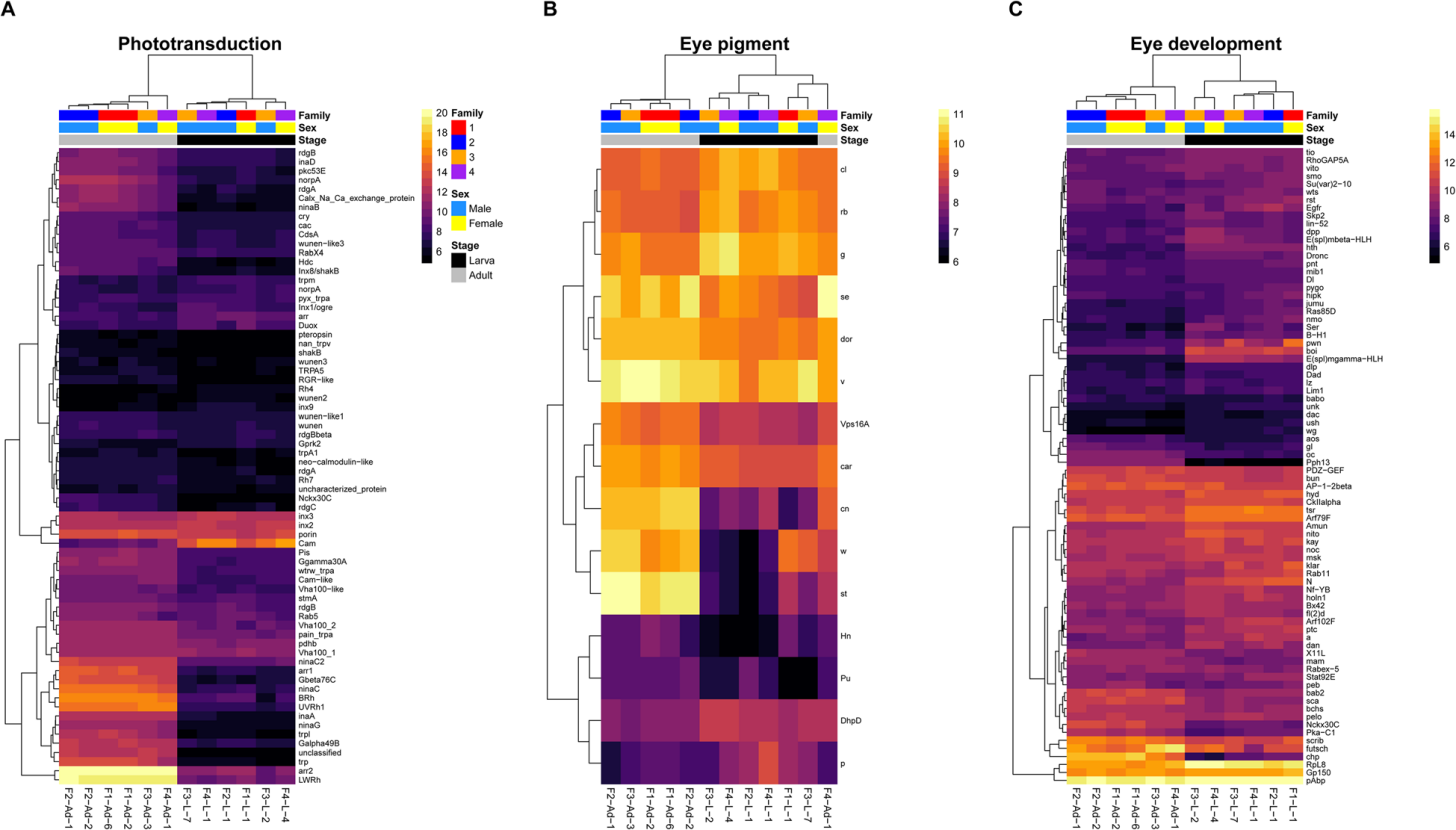
Expression heatmaps for differentially expressed top vision homologs in *B. anynana*. (**A**) Phototransduction genes. (**B**) Eye pigment genes. (**C**) Eye development genes. Counts were normalized by variance stabilizing transformation, with warmer colors indicating higher expression. Rows denote individual genes, and columns denote samples, both of which are clustered by gene expression. Family indicates the family from which the sample was derived, Sex indicates the sex of the sample, and Stage indicates the developmental stage of the sample. Heatmaps were created with pheatmap v1.0.12 [62] in R v3.6.2 [61]

**Fig. 5:**
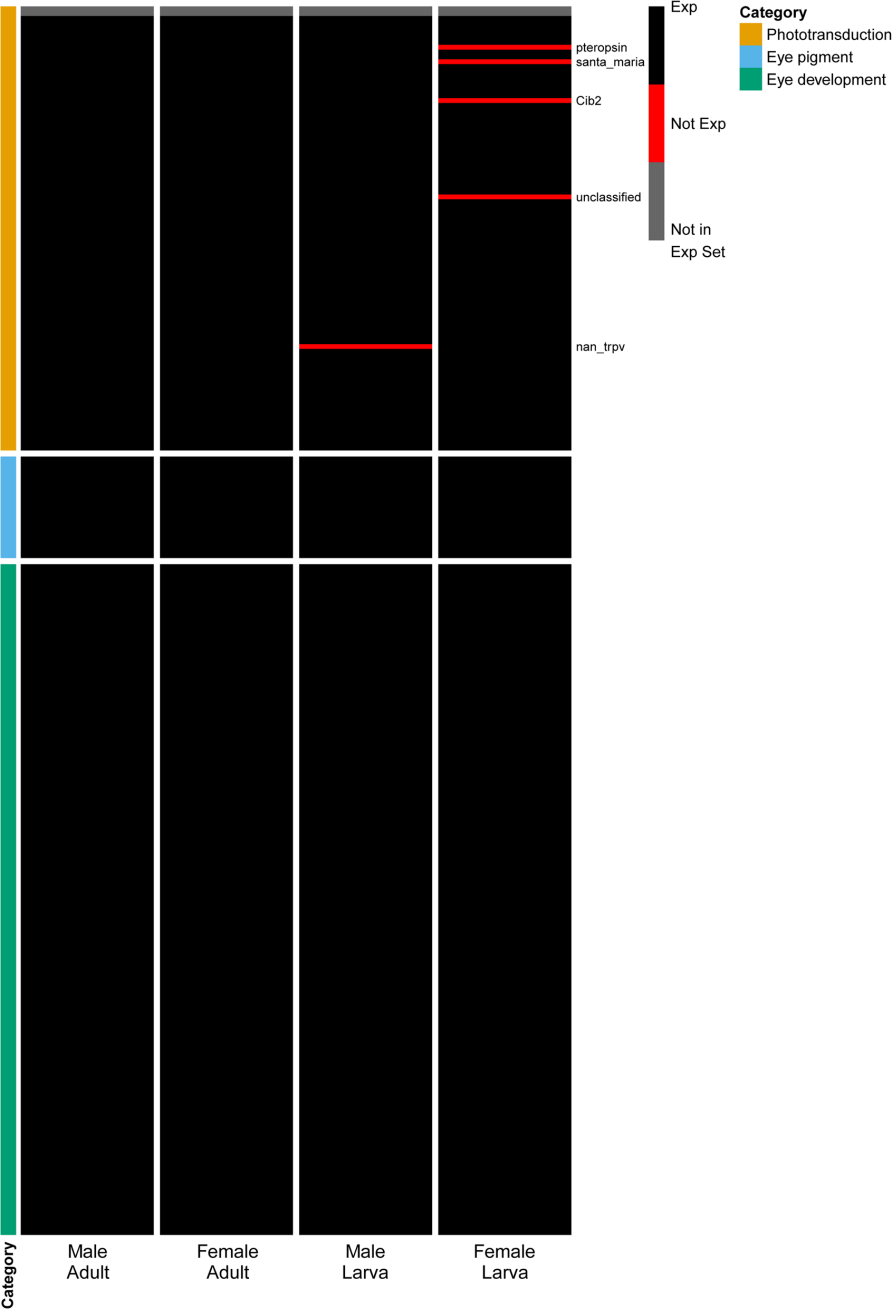
Presence/absence of expression maps for the top vision homologs in *B. anynana*. Rows denote individual genes, and columns denote sample group. Genes that were expressed are indicated in black, while red indicates genes that were not expressed. Genes colored in grey were identified in the *B. anynana* genome but were not present in the head expression set (i.e., not expressed in any group). Category indicates the gene family to which each gene belongs. Expression maps were created with pheatmap v1.0.12 [62] in R v3.6.2 [61]

Blast hits for 252 of the 274 queried vision genes (92 %) resulted in the identification of 1,555 putative homologs in the *B. anynana* genome (Additional file 2: Table S9). Of these homologs, 429 were associated with phototransduction, 76 with eye pigment, and 1,050 with eye development. To identify the top homolog candidates for each of the queried vision genes, we collected the best blast hit, resulting in a set of 252 *B. anynana* vision genes, 250 of which were within the head expression set (Additional file 2: Table S10). Of these top homologs, 165 (65 %) were differentially expressed between larval and adult heads (FDR < 0.05), with 88 (57 phototransduction genes, 10 eye pigment genes, and 21 eye development genes) upregulated in adults and 77 (13 phototransduction genes, 5 eye pigment genes, and 59 eye development genes) upregulated in larvae (Figs. 2 and 4). Only genes associated with phototransduction were significantly enriched in the full differentially expressed gene set (phototransduction, FDR = 1.12 × 10^− 5^; eye pigment, FDR = 0.13; eye development, FDR = 0.26). Out of the 252 identified vision gene homologs, only five phototransduction genes showed evidence of sex-/stage-specific expression, with the expression of *nan_trpv* being absent in male larvae and *Cib2*, *pteropsin*, *santa_maria*, and *unclassified* not showing expression in female larvae (Fig. 5; Additional file 2: Table S11).

**Fig. 6:**
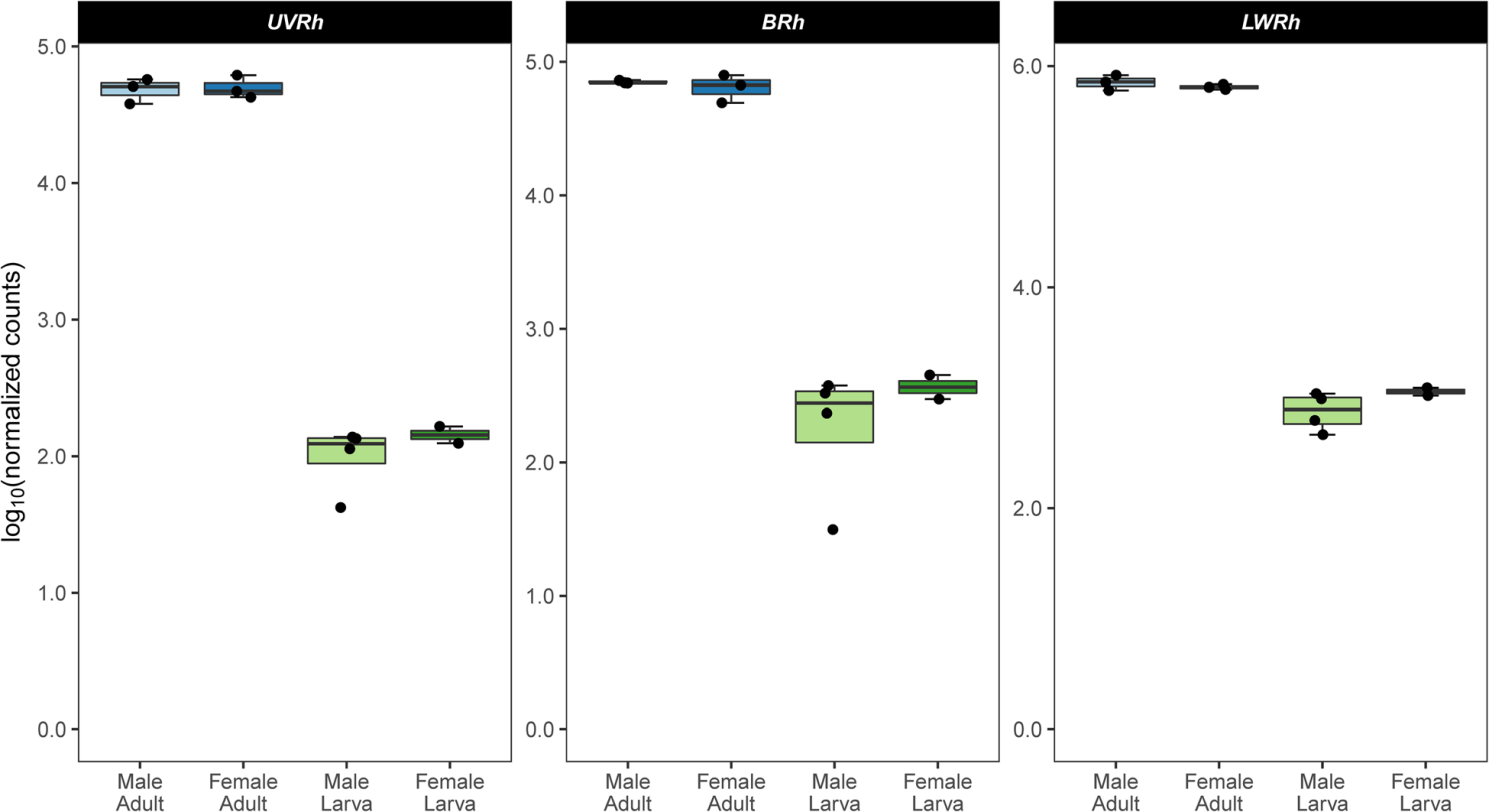
Log transformed normalized counts of visual opsin genes in larvae and adults. All opsins were expressed in both sexes of both stages. Horizontal lines within the boxes denote the median. The upper and lower bounds of the boxes indicate the 25th and 75th percentiles, and whiskers extend to the largest count value ≤ 1.5 × the interquartile range. Y-axes are best fit for each gene. Boxplots were created using ggplot2 v3.3.2 [60] in R v3.6.2 [61]

A total of seven opsins (including three visual opsins: *UVRh*, *BRh*, and *LWRh*) were identified in the expression set. While all seven opsins were expressed in both developmental stages, each was significantly upregulated in adults relative to larvae (*UVRh*, log_2_FC = 8.72, FDR = 1.99 × 10^− 191^; *BRh*, log_2_FC = 7.84, FDR = 1.58 × 10^− 68^; *LWRh*, log_2_FC = 9.37, FDR < 2.22 × 10^− 308^; *Rh7*, log_2_FC = 1.15, FDR = 5.84 × 10^− 4^; *pteropsin*, log_2_FC = 3.65, FDR = 2.84 × 10^− 4^; *unclassified*, log_2_FC = 14.71, FDR = 1.19 × 10^− 52^; *RGR-like*, log_2_FC = 4.96, FDR = 6.07 × 10^− 10^; Fig. 6; Additional file 2: Table S10).

In addition to the *Heliconius melpomene* and *D. melanogaster* vision homologs we identified, manual searches of the Blast2GO functional annotation identified an additional 23 vision-related genes, including numerous genes putatively associated with phototransduction and eye development (Additional file 2: Table S12). Of these genes, 14 were differentially expressed between larval and adult heads, with seven upregulated in adults and seven upregulated in larvae (Additional file 1: Fig. S8).

### Chemosensory genes

#### Odorant binding proteins

**Fig. 7:**
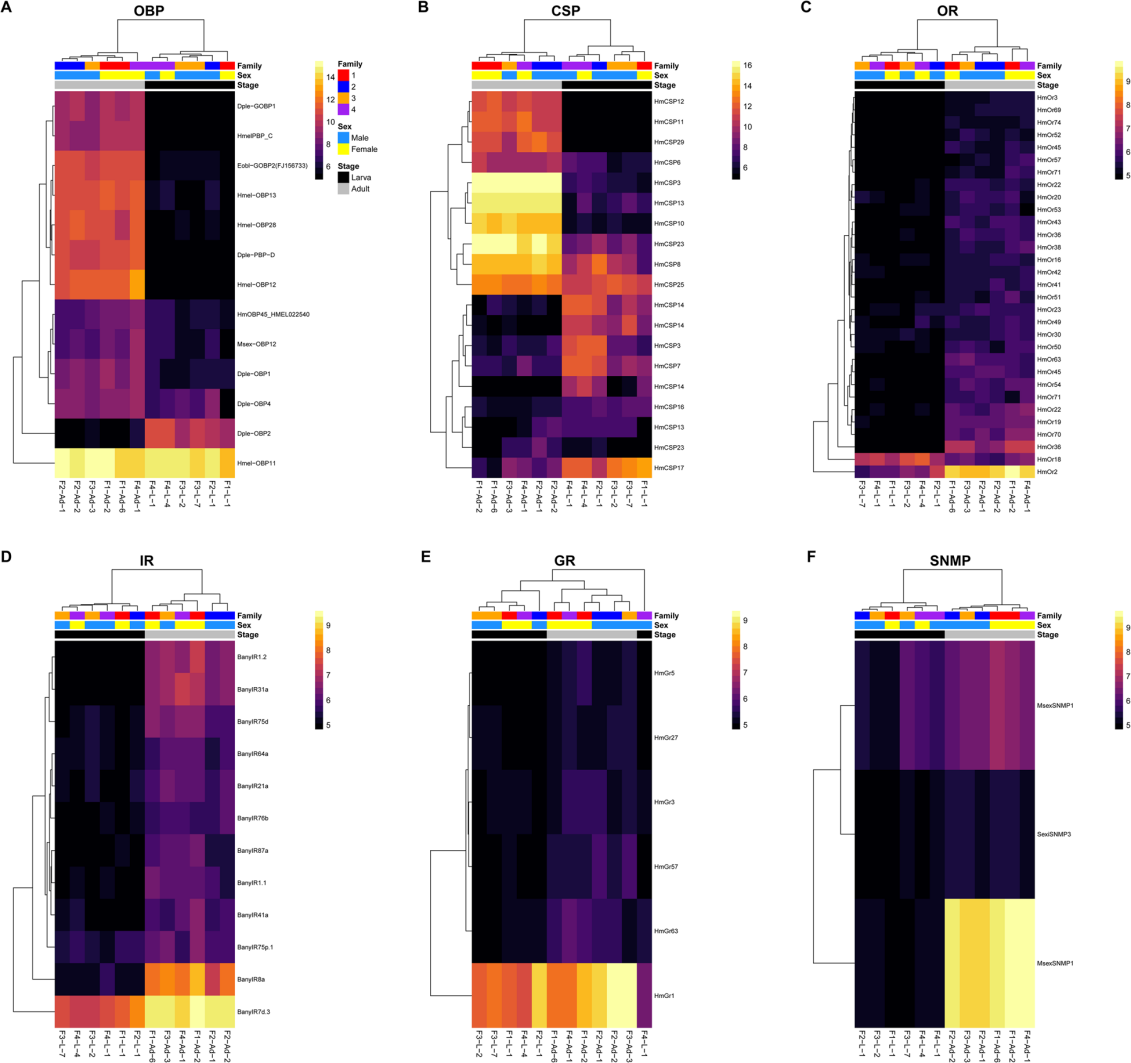
Expression heatmaps for differentially expressed top chemosensory homologs in *B. anynana*. (**A**) Odorant binding proteins. (**B**) Chemosensory proteins. (**C**) Odorant receptors. (**D**) Ionotropic receptors. (**E**) Gustatory receptors. (**F**) Sensory neuron membrane proteins. Counts were normalized by variance stabilizing transformation, with warmer colors indicating higher expression. Rows denote individual genes, and columns denote samples, both of which are clustered by gene expression. Family indicates the family from which the sample was derived, Sex indicates the sex of the sample, and Stage indicates the developmental stage of the sample. Heatmaps were created with pheatmap v1.0.12 [62] in R v3.6.2 [61]

**Fig. 8:**
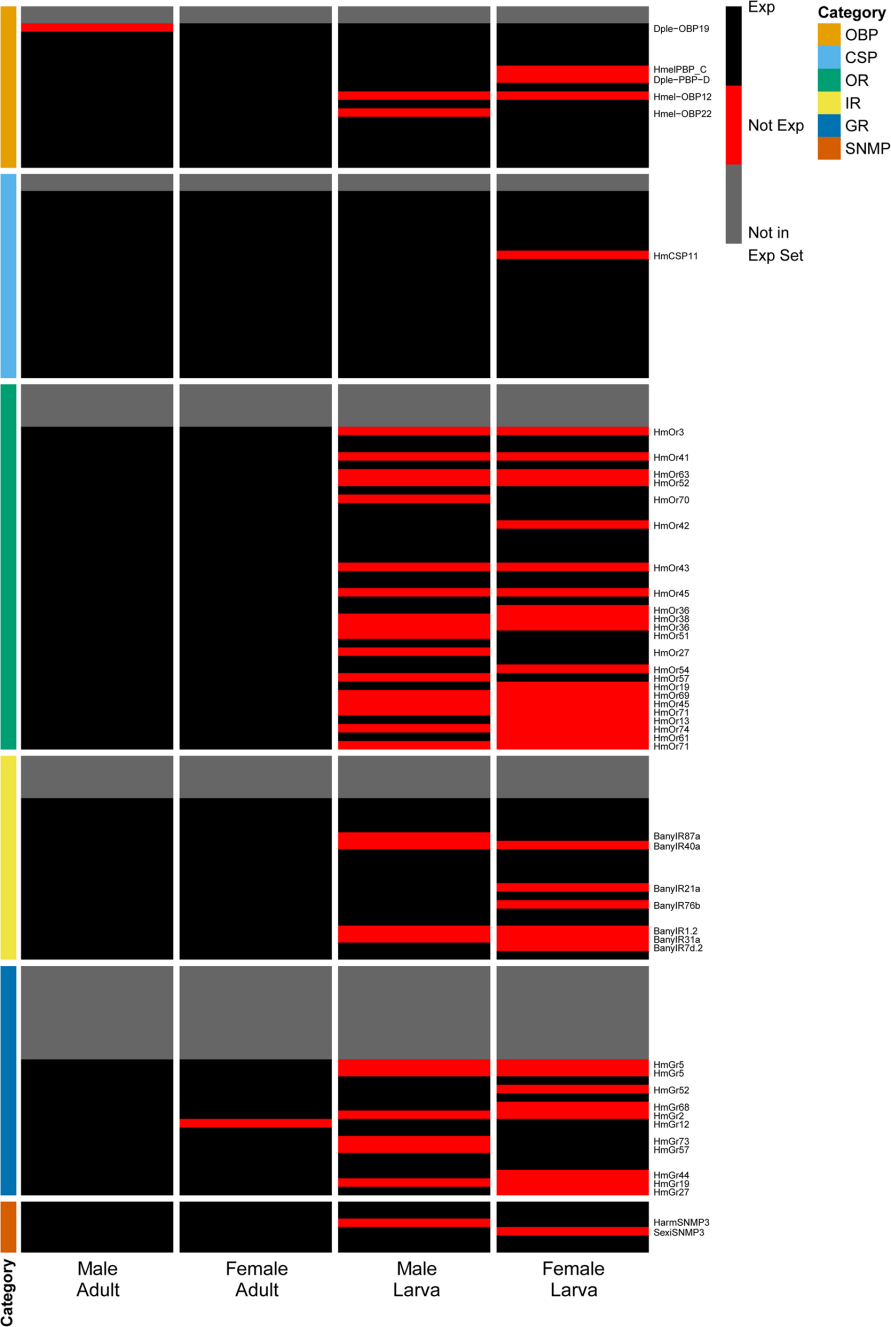
Presence/absence of expression maps for the top chemosensory homologs in *B. anynana*. Rows denote individual genes, and columns denote sample group. Genes that were expressed are indicated in black, while red indicates genes that were not expressed. Genes colored in grey were identified in the *B. anynana* genome but were not present in the head expression set (i.e., not expressed in any group). Category indicates the gene family to which each gene belongs. OBP = odorant binding protein, CSP = chemosensory protein, OR = odorant receptor, IR = ionotropic receptor, GR = gustatory receptor, SNMP = sensory neuron membrane protein. Expression maps were created with pheatmap v1.0.12 [62] in R v3.6.2 [61]

Blast hits for 28 of the 273 queried OBP genes resulted in the identification of 48 putative homologs in the *B. anynana* genome (Additional file 2: Table S13). We retained only those containing pfam01395 or smart00708 domains, which resulted in a set of 19 *B. anynana* OBP genes, 17 being in the head expression set (Additional file 2: Table S14). Of these homologs, 13 (76 %) were differentially expressed (FDR < 0.05), 12 of which were upregulated in adults and one of which was upregulated in larvae (Fig. 7A). OBPs were not significantly enriched in the full differentially expressed gene set (FDR = 0.10). Five OBPs showed stage-/sex-specific expression, including a homolog of *Hmel-OBP12* (*BANY.1.2.g14367*) that was only expressed in the adult stage, and *Dple-OBP19* (*BANY.1.2.g20356*), which was expressed in female but not male adults (Fig. 8; Additional file 2: Table S11). Three other OBPs exhibited sex-specific expression in larvae, with *HmelPBP_C* (*BANY.1.2.g06880*) and *Dple-PBP-D* (*BANY.1.2.g06881*) showing male-specific expression and *Hmel-OBP22* (*BANY.1.2.g19953*) showing female-specific expression.

#### Chemosensory proteins

Blast hits for 18 of the 34 queried CSP genes and one gene identified in our manual search resulted in the identification of 27 putative homologs in the *B. anynana* genome (Additional file 2: Table S15). Twenty-four of these candidate CSP genes contained the pfam03392 domain, 22 of which were in the head expression set (Additional file 2: Table S16). Of these homologs, 19 (86 %) were differentially expressed (FDR < 0.05), with 11 upregulated in adults and eight upregulated in larvae (Fig. 7B). CSPs were significantly enriched in the full differentially expressed gene set (FDR = 3.56 × 10^− 3^). While there was evidence that all CSPs in the expression set were expressed in both life stages, *HmCSP11* (*BANY.1.2.g12993*) was not expressed in female larvae (Fig. 8; Additional file 2: Table S11).

#### Odorant receptors

Blast hits for 38 of the 70 OR genes and four genes found via a manual search resulted in the identification of 50 putative homologs in the *B. anynana* genome (Additional file 2: Table S17). In total, 43 of these were retained as *B. anynana* OR genes by confirmation of the presence of either the pfam02949 or pfam08395 protein domain (Additional file 2: Table S18). Of these homologs, 38 were in the head expression set, 31 (82 %) of which were differentially expressed between larvae and adults (FDR < 0.05). These differentially expressed genes consisted of 30 that were upregulated in adults and one that was upregulated in larvae (Fig. 7C). Additionally, ORs were significantly enriched in the full differentially expressed gene set (FDR = 1.19 × 10^− 3^). Thirteen ORs were found to exhibit stage-specific expression, all of which were expressed only in adults (Fig. 8; Additional file 2: Table S11). Moreover, 10 ORs showed sex-specific expression in larvae, with six only expressed in male heads (*HmOr13*, *HmOr19*, *HmOr36*, *HmOr42*, *HmOr54*, and *HmOr61*) and 4 only expressed in female heads (*HmOr27*, *HmOr51*, *HmOr57*, and *HmOr70*).

#### Ionotropic receptors

We mapped 24 of the 31 *B. anynana* IR sequences from [63] to genes in the reference genome, 19 of which were within the head expression set (Additional file 2: Table S19). Of these homologs, 12 (63 %) were differentially expressed (FDR < 0.05), all of which were upregulated in adults (Fig. 7D). However, IRs were not significantly enriched in the full differentially expressed gene set (FDR = 0.34). Three IRs were found to only be expressed in adult heads: *BanyIR31a*, *BanyIR1.2*, and *BanyIR40a* (Fig. 8; Additional file 2: Table S11). In addition, four IRs were expressed in a sex-specific fashion in larvae, with three only expressed in male larvae (*BanyIR21a*, *BanyIR76b*, and *BanyIR7d.2*) and one only expressed in female larvae (*BanyIR87a*).

#### Gustatory receptors

Blast hits for 24 of the 73 GR genes resulted in the identification of 39 putative homologs in the *B. anynana* genome (Additional file 2: Table S20). We retained only those that contained the pfam08395 domain, resulting in a set of 27 *B. anynana* GR genes, 16 of which were in the head expression set (Additional file 2: Table S21). Of these homologs, six (38 %) were differentially expressed (FDR < 0.05), all of which were upregulated in adults (Fig. 7E). GRs were not significantly enriched in the full differentially expressed gene set (FDR = 0.94). Four GRs, consisting of homologs of *HmGr2*, *HmGr5*, and *HmGr19*, showed adult-specific expression (Fig. 8; Additional file 2: Table S11). Furthermore, one *HmGr12* homolog (*BANY.1.2.g06465*) showed male-specific expression in adults, and six GRs showed sex-specific expression in larvae, with four exhibiting male-specific expression (*HmGr27*, *HmGr44*, *HmGr52*, and *HmGr68*) and two exhibiting female-specific expression (*HmGr57* and *HmGr73*).

#### Sensory neuron membrane proteins

We identified 16 putative SNMP homologs, consisting of blast hits for nine of the 33 SNMP query genes (Additional file 2: Table S22). Filtering these putative homologs for genes that contained the pfam01130 domain and were annotated as SNMP genes in the functional annotation resulted in a set of six *B. anynana* SNMP genes (Additional file 2: Table S23). All of these were within the head expression set, with three (50 %) being differentially expressed between larvae and adults, each of which were upregulated in adults (Fig. 7F). SNMPs, however, were not significantly enriched in the full differentially expressed gene set (FDR = 0.81). Two SNMP homologs showed sex-specific expression in larvae, with *SexiSNMP3* (*BANY.1.2.g07849*) exhibiting male-specific expression and *HarmSNMP3* (*BANY.1.2.g07846*) exhibiting female-specific expression (Fig. 8; Additional file 2: Table S11).

### Developmental and wing patterning genes

**Fig. 9:**
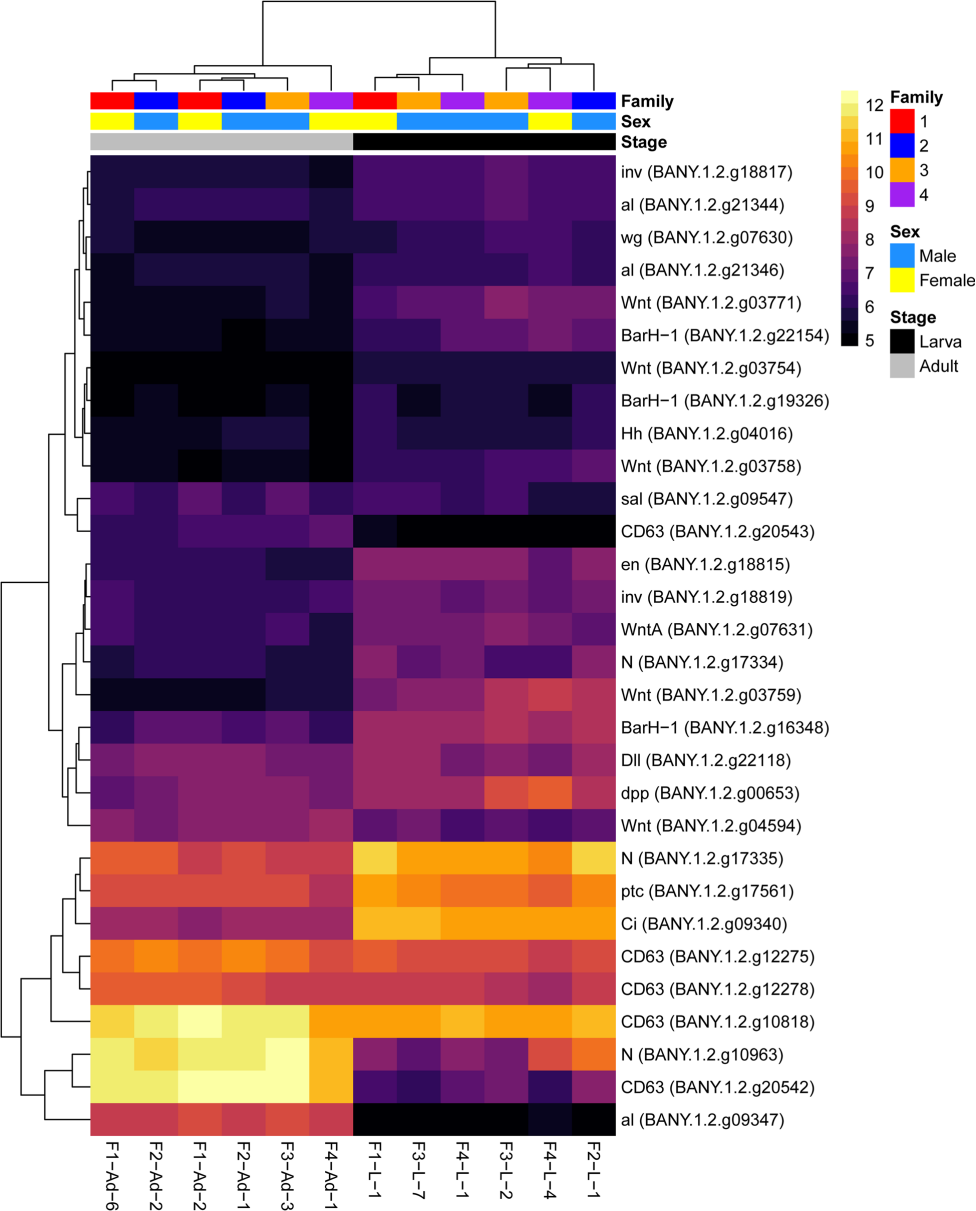
Expression heatmap of differentially expressed genes linked to wing patterning. Counts were normalized by variance stabilizing transformation, with warmer colors indicating higher expression. Rows denote individual genes, and columns denote samples, both of which are clustered by gene expression. Family indicates the family from which the sample was derived, Sex indicates the sex of the sample, and Stage indicates the developmental stage of the sample. This heatmap was created with pheatmap v1.0.12 [62] in R v3.6.2 [61]

A total of 52 genes associated with wing patterning in butterflies were found in the head expression set of larval and adult *B. anynana* (Additional file 2: Table S24; see [64] and [65] for butterfly wing patterning genes). These genes include homologs for *al*, *antp*, *ap*, *BarH-1*, *CD63*, *Ci*, *Dll*, *dpp*, *dsx*, *EcR*, *en*, *Hh*, *inv*, *N*, *optix*, *ptc*, *sal*, *wg*, and several Wnt genes. Of these, 30 (58 %) were differentially expressed between larvae and adults (FDR < 0.05), including homologs for *al*, *BarH-1*, *CD63*, *Ci*, *Dll*, *dpp*, *en*, *Hh*, *inv*, *N*, *ptc*, sal, *wg*, *Wnt, and WntA* (Figs. 2 and 9). Copies of two genes known to be involved with eyespot development in *B. anynana*, *CD63* (*BANY.1.2.g25497*) and *Ci* (*BANY.1.2.g11922*), were found to exhibit expression specific to adult and larval heads, respectively (Additional file 2: Table S11). Additionally, a copy of *BarH-1* (*BANY.1.2.g16347*) exhibited larva-specific expression, while a *Wnt-5* homolog (*BANY.1.2.g04762*) was expressed in all groups except female larvae.

## Discussion

Our analysis of the gene expression profiles of larval and adult *B. anynana* heads revealed considerable differences between the two developmental stages, with > 50 % of all expressed genes showing differential expression. Furthermore, we identified numerous genes involved with vision and chemosensation and elucidated how the expression of these genes, as well as the expression of known wing patterning genes, differs in the head throughout ontogeny and between the sexes. More than 250 *B. anynana* genes putatively linked to vision-related processes were discovered to be expressed in the head, including genes associated with phototransduction as well as eye pigmentation and development. A total of 143 homologs associated with chemosensation were identified, comprising odorant binding proteins, chemosensory proteins, odorant receptors, ionotropic receptors, gustatory receptors, and sensory neuron membrane proteins. In addition, we found 52 genes previously described as butterfly wing patterning genes that were expressed in larval and/or adult heads, including a *WntA* homolog, a gene known to play a significant role in wing pattern development across a diversity of nymphalid species [66, 67]. To our knowledge, this study is the first attempt to characterize the sensory gene repertoire of *Bicyclus* butterflies and provides a promising resource for investigating differences in the sensory biology of larvae and adult butterflies.

### Overall expression differences between larval and adult heads

In larval heads, upregulated genes were linked to developmental processes, including multicellular organism development and the Wnt signaling pathway (Table 2 and Additional file 2: Table S6). Wnt signaling is known to be involved with cell differentiation and proliferation in animals [68, 69]. In butterflies, Wnt genes have been found to be involved with wing patterning [67, 70] and are expressed in various *B. anynana* tissues during embryogenesis, with *Wnt7* and *Wnt11* both expressed in head tissues [71]. In the current study, we found that numerous Wnt genes are expressed in the heads of fifth instar larvae and adults, including *Wnt-1*, *Wnt-5*, *Wnt-6*, *Wnt-10*, *Wnt-11*, and *WntA* homologs, most of which were upregulated in larval heads.

In addition to developmental processes, genes upregulated in larval heads were also enriched for processes linked to gene expression and protein metabolism. Upregulation of genes involved with these functional categories is possibly in part due to the physiological changes taking place in larval head tissues in preparation for pupation and metamorphosis. As with other holometabolous insects, the larval tissues and organs of butterflies undergo degeneration via autophagy and subsequent remodeling during metamorphosis (see [72] for review). Moreover, in *Manduca sexta*, metamorphic cell death is associated with a marked drop in protein synthesis [73]. Therefore, the enrichment of GO terms involved with gene expression and protein metabolism might be indicative of a similar decrease in protein synthesis in *B. anynana*.

In adult heads, chemoreception processes were enriched, with associated GO terms including detection of chemical stimulus involved in sensory perception of smell, olfactory receptor activity, and odorant binding. These results are consistent with elevated chemosensory gene expression in the adult stage of other Lepidoptera [74] and suggest a greater investment in the chemosensory system of adult *B. anynana*. Indeed, the adult stage partakes in numerous behaviors that larvae do not, including courtship/copulation, oviposition, and foraging for fruit. Furthermore, *B. anynana* adult females are known to cue in on pheromones produced by males for mate choice [51, 52, 75], and chemical cues appear to play just as important of a role as visual cues in mate choice for this species. Finally, the ability of flight permits these butterflies to perform these behaviors in a greatly expanded three-dimensional space compared to the larval stage, possibly requiring adults to maintain a more sensitive and sophisticated chemosensory system.

### Insights into larval and adult phototransduction

A large number of vision-related genes were expressed in *B. anynana* heads, most of which were differentially expressed between adults and larvae. These results have implications for furthering our understanding of the differences in the visual capabilities and phototransduction signaling cascade for different life stages of lepidopterans and other holometabolous insects.

The primary visual organs of larval and adult butterflies have disparate morphologies, with larvae possessing two simple eyes consisting of up to six stemmata and adults having two compound eyes consisting of hundreds of ommatidia [76]. Therefore, it is likely that at least some of the observed patterns in vision-related gene expression in larval and adult heads are due to substantial differences in structure and cell composition. Moreover, during metamorphosis in holometabolous insects, the larval stemmata migrate to the adult optic lobe and continue to function as extraretinal photoreceptors [17, 76, 77]. Consequently, the presence of both adult and larval visual structures in *B. anynana* adults might account for a portion of the upregulation observed in vision genes.

Interestingly, the differentially expressed vision genes upregulated in adult heads were dominated by phototransduction genes (65 %), while differentially expressed genes upregulated in larval heads were largely associated with eye development (77 %). A greater emphasis on phototransduction in adults is perhaps not surprising, as a significant proportion of the adult head consists of eye tissue, and optic lobes have been found to comprise nearly 75 % of the butterfly brain [78–80]. In comparison, the stemmata of larvae occupy a considerably smaller proportion of the larval head. The upregulation of genes involved with eye development in late fifth instar larvae suggests that compound eye developmental processes have initiated just prior to pupation.

Several phototransduction genes were upregulated in larvae relative to adults, including a copy of *norpA, wunen2*, and the innexins *ogre*, *inx2*, and *inx3*. *norpA* encodes the protein phospholipase C (PLC), which is involved with diacylglycerol (DAG) production in the *Drosophila* phototransduction cascade [81, 82]. Moreover, Macias-Muñoz et al. (2019) hypothesized that *wunen* plays a similar role in Lepidoptera phototransduction as *lazaro* in *Drosophila*, which is involved in DAG level regulation [83]. Lepidoptera have three copies of *wunen*, and while *wunen2* was upregulated in larval *B. anynana*, the other two (*wunen* and *wunen3*) were upregulated in adults. The observed stage-biased expression of specific *norpA* and *wunen* copies suggests potential differences in DAG regulation throughout development.

Finally, *ogre*, *inx2*, and *inx3* form gap junction channels, all of which are critical to visual transmission. Specifically, *ogre* and *inx3* are necessary for visual synaptic transmission in retinal pigment cells in the compound eyes of *Drosophila*, while *inx2* plays an essential role in laminar glial cells [84]. The fact that these genes are upregulated in larvae suggests the possibility that gap junctions might be either more integral to larval phototransduction or present in greater density in larval eyes. Future functional work should explore this possibility.

Intriguingly, none of the identified vision-related genes, including the visual opsins, were differentially expressed between male and female larvae or male and female adults, a result consistent with previous studies with *B. anynana* [53, 54]. It is also interesting to note that all visual opsins were expressed in larval and adult heads, suggesting that both developmental stages might be capable of perceiving similar wavelengths of light. Future electrophysiological and behavioral studies should explore the spectral sensitivity and behavioral responses of larval and adult *B. anynana* to different light wavelengths.

From the current study, it is not possible to determine which of these genes are expressed in the eyes, brain, and/or other head structures. Numerous studies have localized phototransduction genes in nonvisual tissues, such as the central nervous system [85, 86]. Therefore, future work should investigate tissue-specific expression and determine the expression patterns of these genes in the eyes. While significant efforts have been made to explore butterfly vision in a number of species, usually focusing on opsins, the results of the current study provide a new set of candidate vision genes for *B. anynana* and will help to expand our understanding of lepidopteran adult, as well as larval, vision.

### Insights into larval and adult chemosensation

In the current study, we identified a total of 143 chemosensory genes, most of which showed differential expression between larval and adult *B. anynana* heads. Notably, we discovered numerous OBPs, CSPs, ORs, IRs, GRs, and SNMPs with stage-/sex-specific expression or that displayed differential expression between the developmental stages. Many of these genes share homology with chemosensory genes associated with pheromone detection, host plant recognition, and foraging in other species of Lepidoptera. Because the functions and specificity of chemosensory genes in *B. anynana* are largely unknown, these genes serve as promising targets for further investigation to expand our understanding of chemically mediated behaviors in this species.

A number of the chemosensory genes identified are candidates for pheromone binding and reception. The gene *BANY.1.2.g22938* was upregulated in adult heads and is homologous to *Hmel-OBP13* (alternative name = *HmOBP20*; [87]), which was recently found to possibly be involved with species-specific recognition of pheromones in *Heliconius* butterflies [88]. In addition to the two genes (*BANY.1.2.g06880* and *BANY.1.2.g06881*) sharing sequence homology with *H. melpomene* and *Danaus plexippus* pheromone binding proteins (*HmelPBP_C* and *Dple-PBP-D*, respectively), *BANY.1.2.g22938* might also be involved with pheromone detection in *B. anynana*. Moreover, two putative pheromone receptor homologs (*HmOr3* and *HmOr30*; [87, 89, 90]) were upregulated in adult heads, suggesting that they might also be involved with chemical-mediated mate choice behaviors. Finally, a homolog of *Dple-OBP19* (*BANY.1.2.g20356*) was expressed in female but not male adult heads, indicating a putative role in male sex pheromone (MSP) detection [75].

In addition to the OBP and OR repertoire, we identified two homologs of *SNMP1* (both *MsexSNMP1* homologs) that were upregulated in adult heads. *SNMP1* a protein that forms a complex with pheromone-detecting ORs and an odorant receptor co-receptor (*Orco*; *HmOR2*; identified in the current study as *BANY.1.2.g12855* in *B. anynana*) in insects [30, 31]. *SNMP1* is involved with pheromone detection in both *D. melanogaster* [30, 91] and numerous lepidopteran species [31, 92–95], and it may also play a role in pheromone detection in *B. anynana*.

Several genes were identified as putatively involved with host plant recognition and/or foraging behavior. A homolog of *Eobl-GOBP2* (*BANY.1.2.g06879*), a gene involved with the detection of plant volatiles in the moth *Ectropis obliqua* [96], was upregulated in adult heads. In addition, a homolog of *HmOR49* (*BANY.1.2.g06204*), a putative citral receptor [87, 97], was upregulated in adult heads. Citral is a plant volatile that is present in plant species such as lemongrass and orange [98] and serves as a food attractant for *Bombyx mori* larvae [99] and an oviposition deterrent in the light brown apple moth, *Epiphyas postvittana* [100]. Moreover, electroantennography (EAG) recordings indicated that citral evokes a response in *B. anynana* antennae (Murphy, Joshi, and Westerman, unpublished data). Homologs for two GRs (*HmGr9* and *HmGr57*) that are characterized as putatively being involved with host plant identification via recognition of the plant alkaloid synephrine in *H. melpomene* [24, 88] were also expressed in *B. anynana* heads. These genes might also be involved with host plant recognition in *B. anynana*. Alternatively, they might be involved with detection of *B. anynana*’s adult food source, ripe/rotting fruit, as synephrine, like citral, is present in citrus fruits [101].

Two IRs upregulated in adult *B. anynana*, *BanyIR1.2* and *BanyIR75d*, are putatively involved with host plant searching behavior, as *IR1.2* and *IR75d* are upregulated in antennae of mated females of the moth *Helicoverpa armigera* [63]. In *B. anynana*, the expression of *BanyIR1.2* was specific to adults, consistent with a possible function in oviposition-related behaviors in this species.

Our results also illuminate genes that play potentially important roles in larval chemoreception. One OBP (a homolog for *Dple-OBP2*), eight CSPs (homologs for *HmCSP3*, *HmCSP7*, *HmCSP13*, *HmCSP14*, *HmCSP16*, and *HmCSP17*), and one OR (a *HmOr18* homolog) were upregulated in larval heads relative to adult heads. Previous studies of OBP and CSP gene expression in larvae and adult *H. armigera* discovered six OBP and four CSP genes that are exclusively expressed in larvae antennae and mouthparts, suggesting that OBP and CSP genes may play a role in larval foraging [102]. Moreover, numerous ORs in the moth *Spodoptera littoralis* were found to be tuned to plant volatiles and are involved with larval foraging behavior [103, 104]. Thus, it is possible that the chemosensory genes upregulated in *B. anynana* larvae might play important roles in mediating larval foraging behavior.

### Expression of wing patterning genes

Wing patterning genes have been hypothesized to underlie assortative mating behaviors and ultimately speciation in Lepidoptera through associations with preference for the traits they influence [58, 59]. This might occur in two ways: (1) both the trait and preference are controlled by the same gene; or (2) the genes controlling the trait and preference for that trait are separate but maintained in high linkage disequilibrium (i.e., inherited together) [57, 105, 106]. Empirical evidence for either of these hypotheses, or for the genetic basis of assortative mate preference more broadly, is relatively slim. However, numerous wing patterning genes are known to influence sensory organ and neural processes in other insect species, providing a promising set of candidate genes for exploration. For instance, *optix*, an eye development gene in *Drosophila* [107], has been co-opted to control red pigmentation in the wings of *Heliconius* butterflies [108]. Furthermore, engrailed (*en*), a gene involved with neurogenesis [109], axonal targeting [110], and neuronal cell fate determination [111], is also linked to butterfly eyespot development in *Bicyclus* [112]. Here, we found that numerous genes known to be involved with wing patterning in butterflies were expressed in *B. anynana* heads, possibly in the brain, eyes, or both tissues. If these genes, particularly those involved with eyespot development in *B. anynana*, are linked to preferences for eyespot traits, they might play a role in the great amount of diversity we see in this taxon (80 + species, with many species living in sympatry) [113, 114]. We propose that these wing patterning genes should be investigated as potential drivers of assortative mate preference and speciation in *Bicyclus* butterflies.

### Lack of differential expression of sensory and wing patterning genes between the sexes

It is interesting to note that none of the vision, chemosensory, or wing patterning genes we identified here were differentially expressed between the sexes of either stage. This suggests that developmental stage is likely a larger factor than sex for the expression of these genes in the head. Alternatively, it is also possible that any effects of sex on vision, chemosensory, or wing patterning genes were either too small or too tissue specific to be detected in our data set. For instance, the number of replicates for the sex comparisons was relatively small (Adults: n = 3 males, n = 3 females; Larvae: n = 4 males, n = 2 females); increasing the number of replicates would better capture the biological variability attributed to sex and result in greater power to detect differentially expressed genes. Furthermore, it is possible that increasing tissue specificity (e.g., sequencing the brain neuropils and various sensory tissues separately) might reveal sex-specific gene expression patterns that are obscured when sequencing the whole head.

## Conclusions

In this study, we identified the sensory gene repertoire of the butterfly *B. anynana* and characterized the expression of these genes in larval and adult heads. While visual and chemosensory genes have been explored in many adult Lepidoptera, few studies have investigated the expression of such genes in their larval stages. Our results provide an initial step in elucidating the differences in sensory processing throughout development in butterflies. Moreover, we identified numerous candidate genes for host plant recognition, foraging, and mate choice, including both chemosensory and wing patterning genes expressed in *B. anynana* heads. Future studies should explore the functions of these candidate genes and determine their tissue specificity.

## Methods

### Animals

*Bicyclus anynana*, a nymphalid butterfly native to subtropical Africa, has been maintained in laboratory colonies since 1988. All animals used in this study are descendants of an original population established in Leiden, Netherlands from 80 gravid females that were collected in Malawi [47]. The population at the University of Arkansas was established via the transfer of ~ 1,000 eggs from a population in Singapore to Fayetteville, AR, USA in spring, 2017. Animals were reared in a climate-controlled, USDA-APHIS approved (Permit # P526P-17-00343) greenhouse facility, which was maintained at approximately 27°C, 70 % relative humidity to induce the wet season phenotype in this species [47]. All experiments were conducted between January and March 2019 (sunrise range: 7:06–7:19 am, sunset range: 5:40–7:36 pm) under a 13:11 h light:dark photoperiod. In addition to natural light, the greenhouse was illuminated with full spectrum (including ultraviolet wavelengths) fluorescent lights (lights on: 7:00 am, off: 8:00 pm).

### Experimental design and tissue collection

Four unique families were created by pairing one three-day-old virgin male and one three-day-old virgin female together in a small mesh cage (31.8 cm × 31.8 cm × 31.8 cm) at 8:00 am for at least three hours to ensure that copulation occurred. After visual confirmation that the pair had copulated, the female was removed from the mating cage and isolated in a new large mesh cage (39.9 cm × 39.9 cm × 59.9 cm) containing a corn plant (*Zea mays*) on which to lay eggs and a slice of moistened banana for food. Each female was then given seven days to lay fertilized eggs on the provided corn plant, after which the egg-laden corn plant was transferred to a new small mesh cage (31.8 cm × 31.8 cm × 31.8 cm).

Upon hatching, larvae were reared in their family-specific cages under identical conditions and were fed corn plants *ad libitum*. To ensure that all four families experienced the same environmental conditions within the greenhouse and to control for any potential unforeseen confounding variables associated with cage location, the physical position of each cage was alternated daily. Upon the morning of reaching the late fifth instar stage, which was determined by the stark change in color from tan/brown to green (Fig. 1A), a subset of the larvae from each family was sacrificed by decapitation with RNase-free scissors (n = 6 larvae total). This stage was chosen to ensure that all larvae were as close as possible in development and because it is the final developmental stage prior to pupation. A second subset from each family was allowed to pupate, and newly eclosed adults (Fig. 1B) were sacrificed by decapitation on the morning of emergence (n = 6 adults total). All decapitations were conducted between 9:30 am–12:00 pm, and heads were immediately transferred into RNase-free, low binding 1.5 ml microcentrifuge tubes (Biotix, San Diego, CA, USA), flash-frozen in liquid nitrogen, and transported to the lab for storage at -80°C until they were processed (Additional file 1: Table S25).

### RNA extraction, library preparation, and sequencing

Each frozen head was immersed in pre-chilled RNAlater-ICE (Ambion; Austin, TX, USA) and incubated at -20°C for approximately 16 h prior to tissue processing. After this incubation period, heads were transferred to a dissecting dish filled with RNAlater-ICE, and all residual thoracic tissue was carefully removed with forceps under a dissecting microscope (Zeiss Stemi 508; Jena, Germany), leaving only head tissue. Individual isolated heads (which included antennae and mouthparts) were then disrupted in lysis buffer with an RNase-free, disposable pestle, and small (< 200 nucleotides) and large RNA (> 200 nucleotides) were extracted in separate fractions using the NucleoSpin® miRNA Kit (Macherey-Nagel; Düren, Germany) following the manufacturer’s recommended protocols. RNA purity, concentration, and integrity for each sample were subsequently determined using a NanoDrop 2000 (Thermo Fisher Scientific; Waltham, MA, USA) and TapeStation 2200 (Agilent; Santa Clara, CA, USA).

After confirmation of RNA quality and quantity, a cDNA library for each head was prepared using 500 ng of large RNA as input for the KAPA mRNA HyperPrep Kit (KAPA Biosystems; Wilmington, MA, USA) combined with the KAPA Unique Dual-Indexed Adapter Kit (KAPA Biosystems; Wilmington, MA, USA). The quality of each cDNA library was subsequently verified using a TapeStation 2200 (Agilent; Santa Clara, CA, USA). All libraries (n = 12) were then shipped on dry ice to the University of Chicago Genomics Facility for secondary quality assessment on a 5300 Fragment Analyzer (Agilent; Santa Clara, CA, USA), and 50 base pair (bp) single-end (SE) sequencing was performed on a single lane of a HiSeq 4000 (Illumina; San Diego, CA, USA).

### Animal sexing

All adults were sexed based on apparent sexually dimorphic features, specifically the presence/absence of androconia (the male-specific pheromone organ). Because larvae do not display any obvious sexual dimorphism, we extracted DNA from individuals using the KAPA Express Extract Kit (KAPA Biosystems; Wilmington, MA, USA) and amplified a female-specific W-chromosome microsatellite ([115]; Genbank accession no.: AY785080) using the primers from [116]. PCR products were then visualized by gel electrophoresis, and females were identified by the presence of a band at ~ 185 bp, while males lacked this band (Additional file 1: Fig. S9).

### Functional annotation

Blast2GO v5.2.5 [117] was used to conduct a *de novo* functional annotation of all genes in the most current *B. anynana* reference genome (v1.2; [56]; http://ensembl.lepbase.org/index.html). First, we used BLASTX v2.6.0+ [118] to search the NCBI ‘nr’ protein database (www.ncbi.nlm.nih.gov) and collected the top 10 hits with an E-value < 10^− 3^. These results were then uploaded into Blast2GO, and further functional classification was performed using the InterProScan [119] function within Blast2GO. Finally, the “Mapping” and “Annotation” steps in Blast2GO were performed using the default parameters, and the resulting functional annotation table was exported.

### Differential gene expression analysis

Prior to expression quantification, the quality of the raw reads was assessed using FastQC v0.11.8 (https://www.bioinformatics.babraham.ac.uk/projects/fastqc/), and Illumina adapter sequences were trimmed using Trimmomatic v0.38 [120]. Trimmed reads were then aligned to the *B. anynana* reference genome (v1.2) using STAR v2.7.1a [121] with the default parameters and the “--twopassMode Basic” option. Reads were quantified using the ‘htseq-count’ script from the HTSeq v0.11.2 Python package [122]. Differential gene expression analysis was conducted using the DESeq2 v1.24.0 package [123] in R v3.6.2 [61].

We conducted two separate differential expression analyses. First, the generalized linear model:

$$y\:\sim\:family\:+\:sex\:+\:stage$$

was fit to each gene using a negative binomial distribution, where *y* denotes the response variable (expression), *family* denotes the family to which each individual belongs (family 1–4), *sex* denotes the sex of each animal (male or female), and *stage* denotes the life stage of each individual (larva or adult). Using this design enabled us to contrast the effect of *stage* while controlling for differences in expression associated with lineage and sex. Second, the generalized linear model:

$$y\:\sim\:group$$

was used, where *group* denotes a grouping variable that combines sex and stage (i.e., male larva, female larva, male adult, and female adult). This design permitted us to contrast the effect of sex for each stage (i.e., male larva vs. female larva and male adult vs. female adult). We note the suboptimal number of replicates (n = 2) for female larvae in the male vs. female larvae analysis; however, we opted to perform this analysis in an attempt to identify any sensory genes that show substantial differential expression between the sexes of this stage. Additionally, because a transcriptome-wide comparison between the sexes of caterpillars has never been conducted (to our knowledge), this analysis will also provide preliminary insights into sex-biased gene expression in lepidopteran larvae.

For both analyses, genes with a total read alignment count < 10 were filtered and not included in the differential expression analysis. Gene expression was calculated as the binary log of the expression fold change (log_2_FC), and the *apeglm* method was used for log_2_FC shrinkage to obtain the most accurate estimates of effect size [124]. Finally, genes with a false discovery rate (FDR; [125]) < 0.05 were retained for downstream analysis.

### Gene ontology enrichment analyses

For further characterization, the Fisher’s Exact Test function in Blast2GO was used to test for GO term enrichment. The set of differentially expressed genes identified for the first analysis (*y* ~ *family* + *sex* + *stage* model) was split into genes that showed increased expression in adults (log_2_FC > 0) and those that showed increased expression in larvae (log_2_FC < 0), and each subset was tested separately. For the second analysis (*y ~ group* model), the differentially expressed gene sets were not split due to the small number of genes in each set. The reference set used for all GO enrichment analyses consisted of all genes in the expression set, and only GO terms with an FDR < 0.05 were considered significantly enriched. The list of enriched GO terms for each analysis was then reduced to the most specific terms for visualization. Additionally, the reduced lists of enriched GO terms were processed using REVIGO (http://revigo.irb.hr/; [126]), which further eliminated redundancy and organized GO terms into treemaps consisting of related superclusters.

### Identification of visual genes

To identify genes involved with vision (i.e., phototransduction, eye pigment, and eye development) in *B. anynana*, we first collected the coding sequences (CDS) of 74 putative *H. melpomene* phototransduction genes from [44] and the protein sequences of 200 *D. melanogaster* phototransduction, eye pigment, and eye development genes compiled by [54]. We then used BLASTX and BLASTP (BLAST v2.2.30+; [118]) to query these sequences against the *B. anynana* reference genome proteins in Lepbase (http://blast.lepbase.org/) and identify homologs. Homologs were determined based on hits with an E-value < 1 × 10^− 10^, and the top candidate for each query gene was identified as the hit with the lowest E-value. In cases where numerous hits had identical E-values, ties were broken by selecting the hit with the highest bit score. Finally, to identify additional putative vision genes, we manually searched the Blast2GO annotation descriptions, best blast hits, and GO annotations for terms linked to vision, including: “eye,” “ommatidia,” “ommatidium,” “opsin,” “photoreceptor,” “phototransduction,” “retina,” and “visual”. Fisher’s exact tests were conducted to test if the identified vision-related genes were enriched in the differentially expressed gene set, with all expressed genes as the reference set.

To explore stage-/sex-specific expression, we investigated the normalized counts for individuals in each group (i.e., male larva, female larva, male adult, and female adult). Non-zero counts for any individual(s) within a group for a given gene were considered evidence for expression, while zero counts for all individuals in a group were considered evidence that a given gene was not expressed (note: it is possible that these genes are actually lowly expressed but at levels below the detection threshold for the sequencing depth of the current study).

### Identification of chemosensory genes

To identify genes involved with chemosensation in *B. anynana*, we collected 273 lepidopteran and *D. melanogaster* OBP protein sequences from [45], 34 *H. melpomene* CSP protein sequences from [87], 70 *H. melpomene* OR protein sequences from [87], 31 *B. anynana* IR sequences from [63], 73 *H. melpomene* GR protein sequences from [24], and 33 lepidopteran SNMP sequences from [31]. These sequences were then queried against the *B. anynana* reference genome with BLASTX or BLASTP in Lepbase (http://blast.lepbase.org/) to identify putative homologs. With the exception of the previously identified IR sequences in *B. anynana*, all hits with an E-value < 1 × 10^− 10^ were further screened for conserved protein domains specific to each gene family using CD-Search [127]. Specifically, sequences with hits for the following domains were retained: OBPs, either pfam01395 (PBP/GOBP family) *or* smart00708 (Insect pheromone/odorant binding protein domains); CSPs, pfam03392 (Insect pheromone-binding family, A10/OS-D); ORs, either pfam02949 (7tm Odorant receptor) *or* pfam08395 (7tm Chemosensory receptor); GRs, pfam08395 (7tm Chemosensory receptor); and SNMPs, pfam01130 (CD36 family). Because the CD36 superfamily common to SNMPs consists of three different protein families, only one of which includes SNMPs [128], we filtered the final putative SNMP sequences by only retaining those that were also annotated as SNMPs in our functional annotation.

In addition, we performed a manual search of the Blast2GO functional annotation to identify any additional putative OBP, CSP, OR, IR, GR, and SNMP genes. Specifically, we searched the Blast2GO descriptions and best blast hits for key terms, including: “odorant binding protein,” “pheromone binding protein,” “chemosensory protein,” “ejaculatory bulb-specific protein 3,” “odorant receptor,” “olfactory receptor,” “gustatory receptor,” and “sensory neuron membrane protein” and subjected any putative chemosensory genes to the conserved protein domain filtration described above. Fisher’s exact tests were used to test if the identified chemosensory gene categories were enriched in the differentially expressed gene set, with all expressed genes composing the reference set. P-values resulting from these six tests, along with the p-values resulting from the vision gene enrichment tests, were corrected for multiple comparisons (FDR) [125]. Finally, sex-/stage-specific gene expression was determined as previously described for the vision genes.

### Identification of wing patterning genes

To further explore the expression of genes that might be relevant to sensory processing and signaling during mate choice, we manually searched our *de novo* functional annotation for genes known to be involved with wing patterning, many of which have been hypothesized to also be involved in mate preference as well as other behaviors. These genes included several known *B. anynana* wing patterning genes, such as those coding the proteins Antennapedia (*antp*) [129, 130], apterous (*ap*) [131], CD63 antigen (*CD63*) [132], Cubitus interruptus (*Ci*) [133], decapentaplegic (*dpp*) [134], Distal-less (*Dll*) [135], doublesex (*dsx*) [116], Ecdysone Receptor (*EcR*) [136], Engrailed (*en*) [137], hedgehog (*Hh*) [129], Invected (*inv*) [137], Notch (*N*) [138], patched (*ptc*) [138], Spalt (*sal*) [137], Ultrabithorax (*Ubx*) [130, 133], and wingless (*wg*) [132], as well as genes known to be critical for wing patterning in *Heliconius* and other butterflies, including *aristaless* [139], *BarH-1* [140], *cortex* [141], *optix* [108], and *Wnt* [67, 70].

## Supplementary Information

**Additional file 1:** **Supplemental Tables S1 and S25**; captions for **Supplemental Tables S2-S24**; **Supplemental Figures S1-S9**.

**Additional file 2: Supplemental Tables S2-S24**.

## Data Availability

Raw sequence data associated with this study are accessible through the NCBI Sequence Read Archive (SRA) database under BioProject ID PRJNA730880. All other data presented in this study are available within this manuscript and its additional files.

## Abbreviations

bp: base pair
cDNA: Complementary DNA
CDS: Coding sequence
CSP: Chemosensory protein
DAG: Diacylglycerol
DEGs: Differentially expressed genes
EAG: Electroantennography
FDR: False discovery rate
GO: Gene ontology
GR: Gustatory receptor
GSN: Gustatory sensory neuron
iGluRs: Ionotropic glutamate receptors
IR: Ionotropic receptor
log_2_FC: Log_2_(fold change)
MSP: Male sex pheromone
OBP: Odorant binding protein
OR: Odorant receptor
OSN: Olfactory sensory neuron
PCR: Polymerase chain reaction
PLC: Phospholipase C
RNA-seq: RNA-sequencing
SD: Standard deviation
SE: Single-end
SNMP: Sensory neuron membrane protein

## References

[1] Nevitt GA (2008). Sensory ecology on the high seas: The odor world of the procellariiform seabirds. J Exp Biol.

[2] Fischer S, Oberhummer E, Cunha-Saraiva F, Gerber N, Taborsky B (2017). Smell or vision? The use of different sensory modalities in predator discrimination. Behav Ecol Sociobiol.

[3] Shine R, Webb JK, Lane A, Mason RT (2005). Mate location tactics in garter snakes: effects of rival males, interrupted trails and non-pheromonal cues. Funct Ecol.

[4] Robertson KA, Monteiro A (2005). Female Bicyclus anynana butterflies choose males on the basis of their dorsal UV-reflective eyespot pupils. Proc R Soc B Biol Sci.

[5] Bakker TCM, Mundwiler B (1994). Female mate choice and male red coloration in a natural three-spined stickleback (Gasterosteus aculeatus) population. Behav Ecol.

[6] Chapman FM (1935). The courtship of Gould’s manakin (Manacus vitellinus vitellinus) on Barro Colorado Island, Canal Zone. Bull Am Museum Nat Hist.

[7] Coyne JA, Crittenden AP, Mah K (1994). Genetics of a pheromonal difference contributing to reproductive isolation in Drosophila. Science.

[8] Raymond B, Searle JB, Douglas AE (2001). On the processes shaping reproductive isolation in aphids of the Aphis fabae (Scop.) complex (Aphididae: Homoptera). Biol J Linn Soc.

[9] Pélozuelo L, Meusnier S, Audiot P, Bourguet D, Ponsard S (2007). Assortative mating between European corn borer pheromone races: beyond assortative meeting. PLoS One.

[10] Sutton R, Bolton E, Bartels-Hardege HD, Eswards M, Reish DJ, Hardege JD (2005). Chemical signal mediated premating reproductive isolation in a marine polychaete, Neanthes acuminata (arenaceodentata). J Chem Ecol.

[11] Moore RE (1965). Olfactory discrimination as an isolating mechanism between Peromyscus maniculatus and Peromyscus polionotus. Am Midl Nat.

[12] Nevo E, Bodmer M, Heth G (1976). Olfactory discrimination as an isolating mechanism in speciating mole rats. Experientia.

[13] Laukaitis CM, Critser ES, Karn RC (1997). Salivary androgen-binding protein (abp) mediates sexual isolation in *Mus musculus*. Evolution.

[14] Truman JW, Riddiford LM (1999). The origins of insect metamorphosis. Nature.

[15] Passano LM (1961). The regulation of crustacean metamorphosis. Am. Zoologist.

[16] McMenamin SK, Parichy DM (2013). Metamorphosis in teleosts. Curr Top Dev Biol.

[17] Gilbert C (1994). Form and function of stemmata in larvae of holometabolous insects. Annu Rev Entomol.

[18] Arikawa K (2017). The eyes and vision of butterflies. J Physiol.

[19] Stork NE (2018). How many species of insects and other terrestrial arthropods are there on Earth?. Annu Rev Entomol.

[20] Hardie RC (2001). Phototransduction in Drosophila melanogaster. J Exp Biol.

[21] Benton R (2008). Chemical sensing in Drosophila. Curr Opin in Neurobiol.

[22] Terakita A (2005). The opsins. Genome Biol.

[23] Dahanukar A, Hallem EA, Carlson JR (2005). Insect chemoreception. Curr Opin Neurobiol.

[24] Briscoe AD, Macias-Muñoz A, Kozak KM, Walters JR, Yuan F, Jamie GA (2013). Female behaviour drives expression and evolution of gustatory receptors in butterflies. PLoS Genet.

[25] Vogt RG, Riddiford LM (1981). Pheromone binding and inactivation by moth antennae. Nature.

[26] Pelosi P, Calvello M, Ban L (2005). Diversity of odorant-binding proteins and chemosensory proteins in insects. Chem Senses.

[27] Sato K, Pellegrino M, Nakagawa T, Nakagawa T, Vosshall LB, Touhara K (2008). Insect olfactory receptors are heteromeric ligand-gated ion channels. Nature.

[28] Ha TS, Smith DP (2009). Odorant and pheromone receptors in insects. Front Cell Neurosci.

[29] Hansson BS, Stensmyr MC (2011). Evolution of insect olfaction. Neuron.

[30] Benton R, Vannice KS, Vosshall LB (2007). An essential role for a CD36-related receptor in pheromone detection in Drosophila. Nature.

[31] Zhang HJ, Xu W, Chen QM, Sun LN, Anderson A, Xia QY (2020). A phylogenomics approach to characterizing sensory neuron membrane proteins (SNMPs) in Lepidoptera. Insect Biochem Mol Biol.

[32] Clyne PJ, Warr CG, Carlson JR (2000). Candidate taste receptors in Drosophila. Science.

[33] Moon SJ, Köttgen M, Jiao Y, Xu H, Montell C (2006). A taste receptor required for the caffeine response in vivo. Curr Biol.

[34] Lee Y, Moon SJ, Montell C (2009). Multiple gustatory receptors required for the caffeine response in Drosophila. Proc Natl Acad Sci U S A.

[35] Weiss LA, Dahanukar A, Kwon JY, Banerjee D, Carlson JR (2011). The molecular and cellular basis of bitter taste in Drosophila. Neuron.

[36] Dahanukar A, Foster K, Van der Goes van Naters WM, Carlson JR. A Gr receptor is required for response to the sugar trehalose in taste neurons of Drosophila. Nat Neurosci. 2001;4:1182–6. doi:10.1038/nn765. 10.1038/nn765 11704765

[37] Chyb S, Dahanukar A, Wickens A, Carlson JR (2003). Drosophila Gr5a encodes a taste receptor tuned to trehalose. Proc Natl Acad Sci U S A.

[38] Slone J, Daniels J, Amrein H (2007). Sugar receptors in Drosophila. Curr Biol.

[39] Jones WD, Cayirlioglu P, Kadow IG, Vosshall LB (2007). Two chemosensory receptors together mediate carbon dioxide detection in Drosophila. Nature.

[40] Kwon JY, Dahanukar A, Weiss LA, Carlson JR (2007). The molecular basis of CO2 reception in Drosophila. Proc Natl Acad Sci U S A.

[41] Rytz R, Croset V, Benton R (2013). Ionotropic receptors (IRs): chemosensory ionotropic glutamate receptors in Drosophila and beyond. Insect Biochem Mol Biol.

[42] Zhang YV, Ni J, Montell C (2013). The molecular basis for attractive salt-taste coding in Drosophila. Science.

[43] Koh TW, He Z, Gorur-Shandilya S, Menuz K, Larter NK, Stewart S (2014). The Drosophila IR20a clade of ionotropic receptors are candidate taste and pheromone receptors. Neuron.

[44] Macias-Muñoz A, Rangel Olguin AG, Briscoe AD (2019). Evolution of phototransduction genes in Lepidoptera. Genome Biol Evol.

[45] Vogt RG, Große-Wilde E, Zhou JJ (2015). The Lepidoptera odorant binding protein gene family: gene gain and loss within the GOBP/PBP complex of moths and butterflies. Insect Biochem Mol Biol.

[46] Liu W, Jiang XC, Cao S, Yang B, Wang GR (2018). Functional studies of sex pheromone receptors in Asian corn borer Ostrinia furnacalis. Front Physiol.

[47] Brakefield PM, Reitsma N (1991). Phenotypic plasticity, seasonal climate and the population biology of Bicyclus butterflies (Satyridae) in Malawi. Ecol Entomol.

[48] Koch PB, Brakefield PM, Kesbeke F (1996). Ecdysteroids control eyespot size and wing color pattern in the polyphenic butterfly Bicyclus anynana (Lepidoptera: Satyridae). J Insect Physiol.

[49] Kooi RE, Brakefield PM (1999). The critical period for wing pattern induction in the polyphenic tropical butterfly Bicyclus anynana (Satyrinae). J Insect Physiol.

[50] Prudic KL, Jeon C, Cao H, Monteiro A (2011). Developmental plasticity in sexual roles of butterfly species drives mutual sexual ornamentation. Science.

[51] Costanzo K, Monteiro A (2007). The use of chemical and visual cues in female choice in the butterfly Bicyclus anynana. Proc R Soc B Biol Sci.

[52] Westerman EL, Monteiro A (2013). Odour influences whether females learn to prefer or to avoid wing patterns of male butterflies. Anim Behav.

[53] Everett A, Tong X, Briscoe AD, Monteiro A (2012). Phenotypic plasticity in opsin expression in a butterfly compound eye complements sex role reversal. BMC Evol Biol.

[54] Macias-Munoz A, Smith G, Monteiro A, Briscoe AD (2016). Transcriptome-wide differential gene expression in Bicyclus anynana butterflies: female vision-related genes are more plastic. Mol Biol Evol.

[55] Beldade P, Rudd S, Gruber JD, Long AD (2006). A wing expressed sequence tag resource for Bicyclus anynana butterflies, an evo-devo model. BMC Genomics.

[56] Nowell RW, Elsworth B, Oostra V, Zwaan BJ, Wheat CW, Saastamoinen M (2017). A high-coverage draft genome of the mycalesine butterfly Bicyclus anynana. GigaScience.

[57] Westerman EL (2019). Searching for the genes driving assortative mating. PLoS Biol.

[58] Merrill RM, Rastas P, Martin SH, Melo MC, Barker S, Davey J (2019). Genetic dissection of assortative mating behavior. PLoS Biol.

[59] Kronforst MR, Young LG, Kapan DD, McNeely C, O’Neill RJ, Gilbert LE (2006). Linkage of butterfly mate preference and wing color preference cue at the genomic location of wingless. Proc Natl Acad Sci U S A.

[60] Wickham H (2016). ggplot2: elegant graphics for data analysis.

[61] R Core Team. 2019. R: A language and environment for statistical computing. R Foundation for Statistical Computing, Vienna, Austria. URL https://www.R-project.org/.

[62] Kolde, R. 2019. pheatmap: pretty heatmaps. R package version 1.0.12. https://CRAN.R-project.org/package=pheatmap.

[63] Liu NY, Xu W, Dong SL, Zhu JY, Xu YX, Anderson A (2018). Genome-wide analysis of ionotropic receptor gene repertoire in Lepidoptera with an emphasis on its functions of Helicoverpa armigera. Insect Biochem Mol Biol.

[64] Hanly JJ, Wallbank RWR, McMillan WO, Jiggins CD (2019). Conservation and flexibility in the gene regulatory landscape of heliconiine butterfly wings. Evodevo.

[65] Banerjee TD, Monteiro A (2020). Dissection of larval and pupal wings of Bicyclus anynana butterflies. Methods Protoc.

[66] Mazo-Vargas A, Concha C, Livraghi L, Massardo D, Wallbank RWR, Zhang L (2017). Macroevolutionary shifts of WntA function potentiate butterfly wing-pattern diversity. Proc Natl Acad Sci U S A.

[67] Martin A, Reed RD (2014). Wnt signaling underlies evolution and development of the butterfly wing pattern symmetry systems. Dev Biol.

[68] Logan CY, Nusse R (2004). The Wnt signaling pathway in development and disease. Annu Rev Cell Dev Biol.

[69] Wiese KE, Nusse R, van Amerongen R (2018). Wnt signalling: conquering complexity. Development.

[70] Martin A, Reed RD (2010). Wingless and aristaless2 define a developmental ground plan for moth and butterfly wing pattern evolution. Mol Biol Evol.

[71] Holzem M, Braak N, Brattström O, McGregor AP, Breuker CJ (2019). Wnt gene expression during early embryogenesis in the nymphalid butterfly Bicyclus anynana. Front Ecol Evol.

[72] Tettamanti G, Casartelli M (2019). Cell death during complete metamorphosis. Philos TransR Soc Lond B Biol Sci.

[73] Zakeri Z, Quaglino D, Latham T, Woo K, Lockshin RA (1996). Programmed cell death in the tobacco hornworm, Manduca sexta: alteration in protein synthesis. Microsc Res Tech.

[74] Yang CH, Yang PC, Li J, Yang F, Zhang AB (2016). Transcriptome characterization of Dendrolimus punctatus and expression profiles at different developmental stages. PLoS One.

[75] Nieberding CM, de Vos H, Schneider MV, Lassance JM, Estramil N, Andersson J (2008). The male sex pheromone of the butterfly Bicyclus anynana: towards an evolutionary analysis. PLoS One.

[76] Ichikawa T (1991). Integration of colour signals in the medulla of the swallowtail butterfly larva. J Exp Biol.

[77] Briscoe AD, White RH (2005). Adult stemmata of the butterfly Vanessa cardui express UV and green opsin mRNAs. Cell Tissue Res.

[78] Ali FA (1974). Structure and metamorphosis of the brain and suboesophageal ganglion of Pieris brassicae (L.) (Lepidoptera: Pieridae). Trans R Entomol Soc London.

[79] Sivinski J (1989). Mushroom body development in nymphalid butterflies: a correlate of learning?. J Insect Behav.

[80] Heinze S, Reppert SM (2012). Anatomical basis of sun compass navigation I: the general layout of the monarch butterfly brain. J Comp Neurol.

[81] Bloomquist BT, Shortridge RD, Schneuwly S, Perdew M, Montell C, Steller H (1988). Isolation of a putative phospholipase C gene of Drosophila, norpA, and its role in phototransduction. Cell.

[82] Lee YJ, Shah S, Suzuki E, Zars T, O’Day PM, Hyde DR (1994). The Drosophila dgq gene encodes a G alpha protein that mediates phototransduction. Neuron.

[83] Garcia-Murillas I, Pettitt T, Macdonald E, Okkenhaug H, Georgiev P, Trivedi D (2006). lazaro encodes a lipid phosphate phosphohydrolase that regulates phosphatidylinositol turnover during Drosophila phototransduction. Neuron.

[84] Han Y, Xiong L, Xu Y, Tian T, Wang T (2017). The β-alanine transporter BalaT is required for visual neurotransmission in Drosophila. Elife.

[85] Kingston ACN, Cronin TW (2015). Short- and long-wavelength-sensitive opsins are involved in photoreception both in the retina and throughout the central nervous system of crayfish. J Comp Physiol A Neuroethol Sens Neural Behav Physiol.

[86] Donohue MW, Carleton KL, Cronin TW (2017). Opsin expression in the central nervous system of the mantis shrimp Neogonodactylus oerstedii. Biol Bull.

[87] Heliconius Genome Consortium (2012). Butterfly genome reveals promiscuous exchange of mimicry adaptations among species. Nature.

[88] van Schooten B, Meléndez-Rosa J, van Belleghem SM, Jiggins CD, Tan JD, McMillan WO (2020). Divergence of chemosensing during the early stages of speciation. Proc Natl Acad Sci U S A.

[89] Nakagawa T, Sakurai T, Nishioka T, Touhara K (2005). Insect sex-pheromone signals mediated by specific combinations of olfactory receptors. Science.

[90] Wanner KW, Anderson AR, Trowell SC, Theilmann DA, Robertson HM, Newcomb RD (2007). Female-biased expression of odourant receptor genes in the adult antennae of the silkworm, Bombyx mori. Insect Mol Biol.

[91] Jin X, Ha TS, Smith DP (2008). SNMP is a signaling component required for pheromone sensitivity in Drosophila. Proc Natl Acad Sci U S A.

[92] Rogers ME, Sun M, Lerner MR, Vogt RG (1997). Snmp-1, a novel membrane protein of olfactory neurons of the silk moth Antheraea polyphemus with homology to the CD36 family of membrane proteins. J Biol Chem.

[93] Rogers ME, Krieger J, Vogt RG (2001). Antennal SNMPs (sensory neuron membrane proteins) of Lepidoptera define a unique family of invertebrate CD36-like proteins. J Neurobiol.

[94] Rogers ME, Steinbrecht RA, Vogt RG (2001). Expression of SNMP-1 in olfactory neurons and sensilla of male and female antennae of the silkmoth Antheraea polyphemus. Cell Tissue Res.

[95] Krieger J, Raming K, Dewer YME, Bette S, Conzelmann S, Breer H (2002). A divergent gene family encoding candidate olfactory receptors of the moth Heliothis virescens. Eur J Neurosci.

[96] Zhang YL, Fu XB, Cui HC, Zhao L, Yu JZ, Li HL (2018). Functional characteristics, electrophysiological and antennal immunolocalization of general odorant-binding protein 2 in tea geometrid, Ectropis oblique. Int J Mol Sci.

[97] Jordan MD, Anderson A, Begum D, Carraher C, Authier A, Marshall SDG (2009). Odorant receptors from the light brown apple moth (Epiphyas postvittana) recognize important volatile compounds produced by plants. Chem Senses.

[98] Martins P, Sbaite P, Benites C, Wolf Maciel M (2011). Thermal characterization of orange, lemongrass, and basil essential oils. Chem Eng Trans.

[99] Hamamura Y, Naito KI (1961). Food selection by silkworm larvæ, Bombyx mori: Citral, linalyl acetate, linalol, and terpinyl acetate as attractants of larvæ. Nature.

[100] Suckling DM, Karg G, Gibb AR, Bradley SJ (1996). Electroantennogram and oviposition responses of Epiphyas postvittana (Lepidoptera: Tortricidae) to plant volatiles. New Zeal J Crop Hortic Sci.

[101] Stewart I, Newhall WF, Edwards GJ (1964). The isolation and identification of l-synephrine in the leaves and fruit of citrus. J Biol Chem.

[102] Chang H, Ai D, Zhang J, Dong S, Liu Y, Wang G (2017). Candidate odorant binding proteins and chemosensory proteins in the larval chemosensory tissues of two closely related noctuidae moths, Helicoverpa armigera and H. assulta. PLoS One.

[103] de Fouchier A, Walker WB, Montagné N, Steiner C, Binyameen M, Schlyter F (2017). Functional evolution of Lepidoptera olfactory receptors revealed by deorphanization of a moth repertoire. Nat Commun.

[104] de Fouchier A, Sun X, Caballero-Vidal G, Travaillard S, Jacquin-Joly E, Montagné N (2018). Behavioral effect of plant volatiles binding to Spodoptera littoralis larval odorant receptors. Front Behav Neurosci.

[105] Servedio MR (2009). The role of linkage disequilibrium in the evolution of premating isolation. Heredity (Edinb).

[106] Smadja C, Butlin RK (2009). On the scent of speciation: the chemosensory system and its role in premating isolation. Heredity (Edinb).

[107] Seimiya M, Gehring WJ (2000). The Drosophila homeobox gene optix is capable of inducing ectopic eyes by an eyeless-independent mechanism. Development.

[108] Reed RD, Papa R, Martin A, Hines HM, Counterman BA, Pardo-Diaz C (2011). optix drives the repeated convergent evolution of butterfly wing pattern mimicry. Science.

[109] Patel NH, Martin-Blanco E, Coleman KG, Poole SJ, Ellis MC, Kornberg TB (1989). Expression of engrailed proteins in arthropods, annelids, and chordates. Cell.

[110] Whitington PM, Meier T, King P (1991). Segmentation, neurogenesis and formation of early axonal pathways in the centipede, Ethmostigmus rubripes (Brandt). Roux’s Arch Dev Biol.

[111] Condron BG, Patel NH, Zinn K (1994). Engrailed controls glial/neuronal cell fate decisions at the midline of the central nervous system. Neuron.

[112] Keys DN, Lewis DL, Selegue JE, Pearson BJ, Goodrich LV, Johnson RL (1999). Recruitment of a hedgehog regulatory circuit in butterfly eyespot evolution. Science.

[113] Condamin M (1973). Monographie de genre Bicyclus (Lepidoptera Satyridae).

[114] Kodandaramaiah U, Lees DC, Müller CJ, Torres E, Karanth KP, Wahlberg N (2010). Phylogenetics and biogeography of a spectacular Old World radiation of butterflies: the subtribe Mycalesina (Lepidoptera: Nymphalidae: Satyrini). BMC Evol Biol.

[115] Van’t Hof AE, Zwaan BJ, Saccheri IJ, Daly D, Bot ANM, Brakefield PM (2005). Characterization of 28 microsatellite loci for the butterfly Bicyclus anynana. Mol Ecol Notes.

[116] Prakash A, Monteiro A (2020). Doublesex mediates the development of sex-specific pheromone organs in Bicyclus butterflies via multiple mechanisms. Mol Biol Evol.

[117] Conesa A, Götz S, García-Gómez JM, Terol J, Talón M, Robles M (2005). Blast2GO: A universal tool for annotation, visualization and analysis in functional genomics research. Bioinformatics.

[118] Altschul SF, Gish W, Miller W, Myers EW, Lipman DJ (1990). Basic local alignment search tool. J Mol Biol.

[119] Jones P, Binns D, Chang HY, Fraser M, Li W, McAnulla C (2014). InterProScan 5: genome-scale protein function classification. Bioinformatics.

[120] Bolger AM, Lohse M, Usadel B (2014). Trimmomatic: a flexible trimmer for Illumina sequence data. Bioinformatics.

[121] Dobin A, Davis CA, Schlesinger F, Drenkow J, Zaleski C, Jha S (2013). STAR: ultrafast universal RNA-seq aligner. Bioinformatics.

[122] Anders S, Pyl PT, Huber W (2015). HTSeq–a Python framework to work with high-throughput sequencing data. Bioinformatics.

[123] Love MI, Huber W, Anders S (2014). Moderated estimation of fold change and dispersion for RNA-seq data with DESeq2. Genome Biol..

[124] Zhu A, Ibrahim JG, Love MI (2019). Heavy-tailed prior distributions for sequence count data: removing the noise and preserving large differences. Bioinformatics..

[125] Benjamini Y, Hochberg Y (1995). Controlling the false discovery rate: a practical and powerful approach to multiple testing. J R Stat Soc Ser B.

[126] Supek F, Bošnjak M, Škunca N, Šmuc T (2011). REVIGO summarizes and visualizes long lists of gene ontology terms. PLoS One.

[127] Marchler-Bauer A, Bryant SH (2004). CD-Search: protein domain annotations on the fly. Nucleic Acids Res.

[128] Vogt RG, Miller NE, Litvack R, Fandino RA, Sparks J, Staples J (2009). The insect SNMP gene family. Insect Biochem Mol Biol.

[129] Saenko SV, Marialva MS, Beldade P (2011). Involvement of the conserved Hox gene Antennapedia in the development and evolution of a novel trait. Evodevo.

[130] Matsuoka Y, Monteiro A (2021). Hox genes are essential for the development of eyespots in Bicyclus anynana butterflies. Genetics.

[131] Prakash A, Monteiro A (2018). *apterous A* specifies dorsal wing patterns and sexual traits in butterflies. Proc R Soc B Biol Sci.

[132] Özsu N, Chan QY, Chen B, Gupta MD, Monteiro A (2017). Wingless is a positive regulator of eyespot color patterns in Bicyclus anynana butterflies. Dev Biol.

[133] Monteiro A, Prudic KL (2010). Multiple approaches to study color pattern evolution in butterflies. Trends Evol Biol.

[134] Connahs H, Tlili S, van Creij J, Loo TYJ, Banerjee TD, Saunders TE (2019). Activation of butterfly eyespots by Distal-less is consistent with a reaction-diffusion process. Development.

[135] Monteiro A, Chen B, Ramos DM, Oliver JC, Tong X, Guo M (2013). Distal-less regulates eyespot patterns and melanization in Bicyclus butterflies. J Exp Zool Part B Mol Dev Evol.

[136] Bhardwaj S, Prudic KL, Bear A, Dasgupta M, Wasik BR, Tong X (2018). Sex differences in 20-hydroxyecdysone hormone levels control sexual dimorphism in Bicyclus anynana wing patterns. Mol Biol Evol.

[137] Monteiro A, Glaser G, Stockslager S, Glansdorp N, Ramos D (2006). Comparative insights into questions of lepidopteran wing pattern homology. BMC Dev Biol.

[138] Beldade P, Peralta CM (2017). Developmental and evolutionary mechanisms shaping butterfly eyespots. Curr Opin Insect Sci..

[139] Westerman EL, VanKuren NW, Massardo D, Tenger-Trolander A, Zhang W, Hill RI (2018). Aristaless controls butterfly wing color variation used in mimicry and mate choice. Curr Biol.

[140] Woronik A, Tunström K, Perry MW, Neethiraj R, Stefanescu C, Celorio-Mancera MP (2019). A transposable element insertion is associated with an alternative life history strategy. Nat Commun.

[141] Nadeau NJ, Pardo-Diaz C, Whibley A, Supple MA, Saenko SV, Wallbank RWR (2016). The gene cortex controls mimicry and crypsis in butterflies and moths. Nature..

