# Supplementary material for: Stage- and sex-specific transcriptome analyses reveal distinctive sensory gene expression patterns in a butterfly
Source: BMC Genomics. 2021 Aug 2;22:584. doi: 10.1186/s12864-021-07819-4 (PMC8327453; doi:10.1186/s12864-021-07819-4)

**Title:** Stage- and sex-specific transcriptome analyses reveal distinctive sensory gene expression patterns in a butterfly

**Authors:** David A. Ernst and Erica L. Westerman

**Supplemental Tables**

**Table S1:** Summary statistics for sequence data (± SD). Mean mapped reads indicates the number of trimmed reads per sample that mapped to the *B. anynana* reference genome (v1.2). Mean reads for DE indicates the number of reads per sample used for differential expression analysis, defined by reads that mapped to genes that were overlapped by at least 10 reads across all libraries.

| **Stage** | **n** | **Total raw**  **reads (×10^6^)** | **Mean raw**  **reads (×10^6^)** | **Mean mapped**  **reads (×10^6^)** | **Mean reads for DE (×10^6^)** |
| --- | --- | --- | --- | --- | --- |
| Larva | 6 | 187.1 | 31.2 ± 2.9 | 27.1 ± 2.1 | 13.0 ± 1.3 |
| Adult | 6 | 200.9 | 33.5 ± 8.5 | 29.6 ± 7.4 | 11.4 ± 2.8 |
| All Stages | 12 | 388.0 | 32.3 ± 6.2 | 28.3 ± 5.3 | 12.2 ± 2.3 |

**Table S2:** Blast2GO functional annotation of *Bicyclus anynana* genome assembly (v1.2) and DESeq2 results from the *y* ~ *family* + *sex* + *stage* model. Genes that were not in the expression set are denoted with “NA” across all DESeq2 results (baseMean, log2FoldChange , lfcSE, pvalue, and padj). See Additional_file_2.

**Table S3:** GO enrichment analyses results for genes upregulated in the adult heads. See Additional_file_2.

**Table S4:** GO enrichment analysis results reduced to the most specific terms for genes upregulated in the adult heads. See Additional_file_2.

**Table S5:** GO enrichment analysis results for genes upregulated in the larva heads. See Additional_file_2.

**Table S6:** GO enrichment analysis results reduced to the most specific terms for genes upregulated in the larva heads. See Additional_file_2.

**Table S7:** Differentially expressed genes between male and female adults. See Additional_file_2.

**Table S8:** Differentially expressed genes between male and female larvae. See Additional_file_2.

**Table S9:** All putative vision homologs identified in *B. anynana* genome assembly (v1.2). Evalue, Bitscore, Perc_Ident, and Perc_Qcov_Per_Subj are reported for the top HSP. See Additional_file_2.

**Table S10:** Top vision homologs (determined by best blast hit) identified in *B. anynana* genome assembly (v1.2). Evalue, Bitscore, Perc_Ident, and Perc_Qcov_Per_Subj are reported for the top HSP. See Additional_file_2.

**Table S11:** Presence/absence expression matrix for vision, chemosensory, and wing patterning genes that show stage- and/or sex-specific expression. See Additional_file_2.

**Table S12:** Additional putative vision-related genes identified via a manual search of the Blast2GO functional annotation. See Additional_file_2.

**Table S13:** All putative odorant binding protein homologs identified in *B. anynana* genome assembly (v1.2). Evalue, Bitscore, Perc_Ident, and Perc_Qcov_Per_Subj are reported for the top HSP. See Additional_file_2.

**Table S14:** Top odorant binding protein homologs (determined by CD-Search) in *B. anynana* genome assembly (v1.2). Evalue, Bitscore, Perc_Ident, and Perc_Qcov_Per_Subj are reported for the top HSP. See Additional_file_2.

**Table S15:** All putative chemosensory protein homologs identified in *B. anynana* genome assembly (v1.2). Evalue, Bitscore, Perc_Ident, and Perc_Qcov_Per_Subj are reported for the top HSP. See Additional_file_2.

**Table S16:** Top chemosensory protein homologs (determined by CD-Search) in *B. anynana* genome assembly (v1.2). Evalue, Bitscore, Perc_Ident, and Perc_Qcov_Per_Subj are reported for the top HSP. See Additional_file_2.

**Table S17:** All putative odorant receptor homologs identified in *B. anynana* genome assembly (v1.2). Evalue, Bitscore, Perc_Ident, and Perc_Qcov_Per_Subj are reported for the top HSP. See Additional_file_2.

**Table S18:** Top odorant receptor homologs (determined by CD-Search) in *B. anynana* genome assembly (v1.2). Evalue, Bitscore, Perc_Ident, and Perc_Qcov_Per_Subj are reported for the top HSP. See Additional_file_2.

**Table S19:** All ionotropic receptor homologs identified in *B. anynana* genome assembly (v1.2). Evalue, Bitscore, Perc_Ident, and Perc_Qcov_Per_Subj are reported for the top HSP. See Additional_file_2.

**Table S20:** All putative gustatory receptor homologs identified in *B. anynana* genome assembly (v1.2). Evalue, Bitscore, Perc_Ident, and Perc_Qcov_Per_Subj are reported for the top HSP. See Additional_file_2.

**Table S21:** Top gustatory receptor homologs (determined by CD-Search) in *B. anynana* genome assembly (v1.2). Evalue, Bitscore, Perc_Ident, and Perc_Qcov_Per_Subj are reported for the top HSP. See Additional_file_2.

**Table S22:** All putative sensory neuron membrane protein homologs identified in *B. anynana* genome assembly (v1.2). Evalue, Bitscore, Perc_Ident, and Perc_Qcov_Per_Subj are reported for the top HSP. See Additional_file_2.

**Table S23:** Top sensory neuron membrane protein homologs (determined by CD-Search) in *B. anynana* genome assembly (v1.2). Evalue, Bitscore, Perc_Ident, and Perc_Qcov_Per_Subj are reported for the top HSP. See Additional_file_2.

**Table S24:** Wing patterning gene homologs identified in the expression set. See Additional_file_2.

**Table S25:** *B. anynana* sample characteristics.

|  | Family 1 | Family 2 | Family 3 | Family 4 | Total |
| --- | --- | --- | --- | --- | --- |
| Male Larva | - | 1 | 2 | 1 | 4 |
| Female Larva | 1 | - | - | 1 | 2 |
| Male Adult | - | 2 | 1 | - | 3 |
| Female Adult | 2 | - | - | 1 | 3 |
| Total | 3 | 3 | 3 | 3 | 12 |

**Supplemental Figures**

**Fig. S1: PCA plot of PC1 and PC2 based on variance stabilized counts for all samples.**


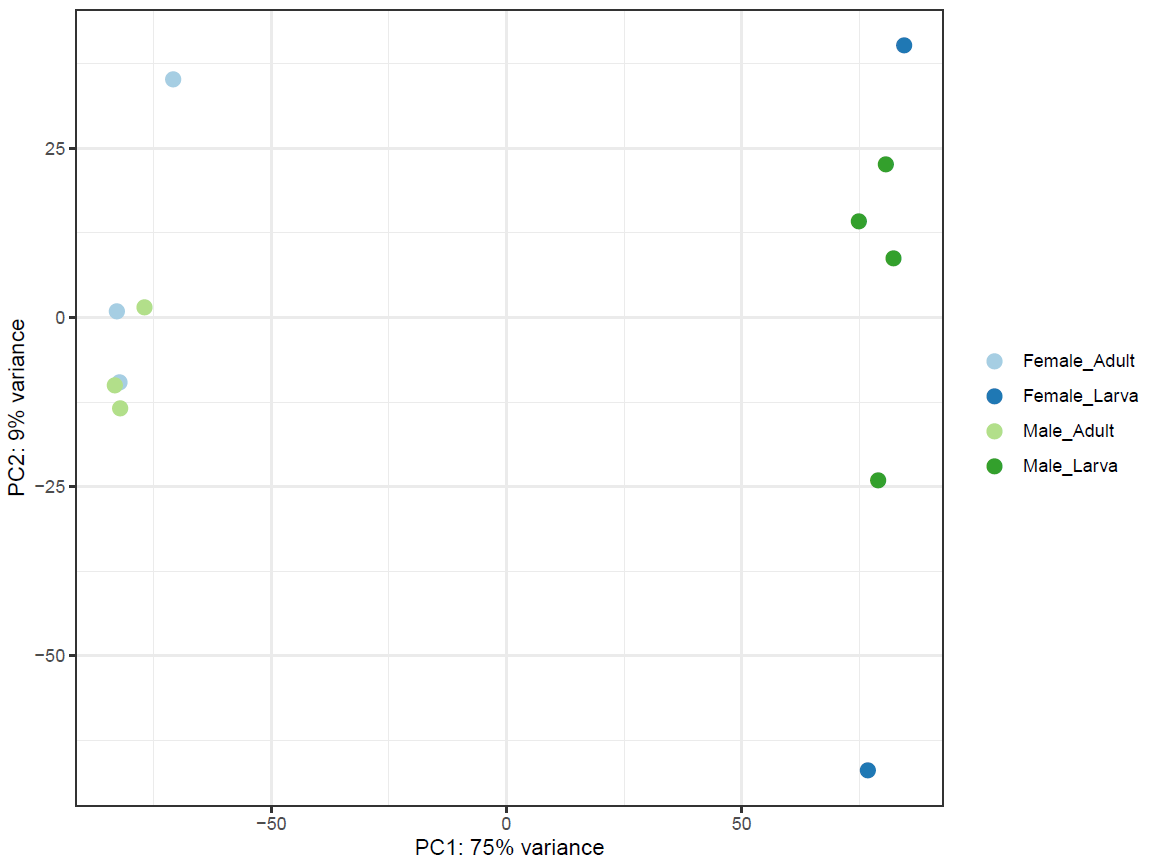


**Fig. S2:** Biological Process treemap summarizing GO terms upregulated in adults. Enriched GO terms were reduced to the most specific terms in Blast2GO, and redundancy was further reduced using REVIGO. The size of each individual rectangle is relative to the absolute value of log_10_(FDR) for each GO term. a) Superclusters of related GO terms. b) All GO term cluster representatives.


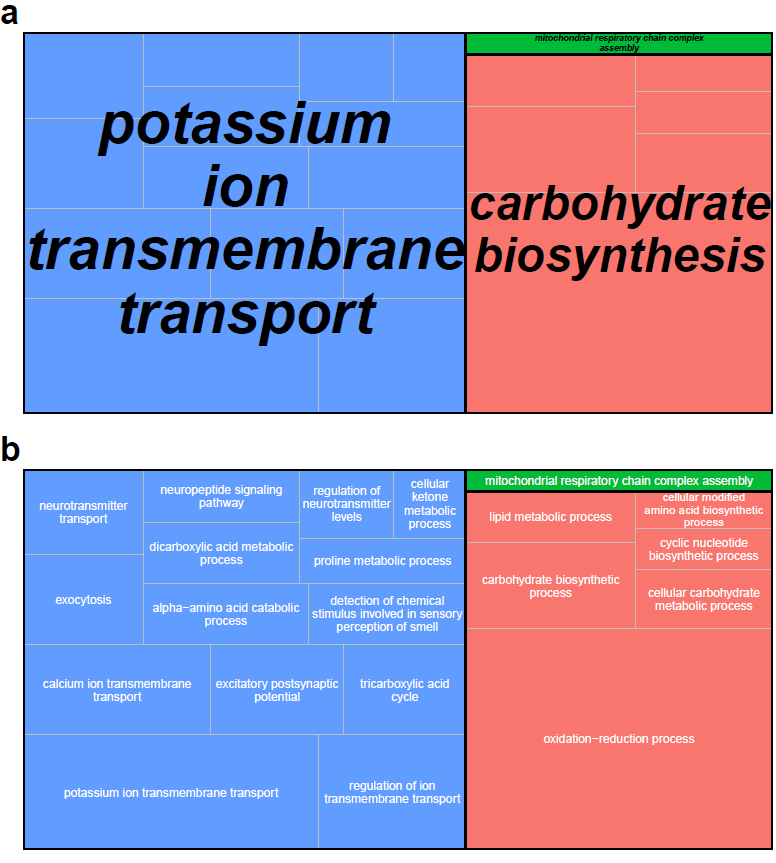


**Fig. S3:** Molecular Function treemap summarizing GO terms upregulated in adults. Enriched GO terms were reduced to the most specific terms in Blast2GO, and redundancy was further reduced using REVIGO. The size of each individual rectangle is relative to the absolute value of log_10_(FDR) for each GO term. a) Superclusters of related GO terms. b) All GO term cluster representatives.


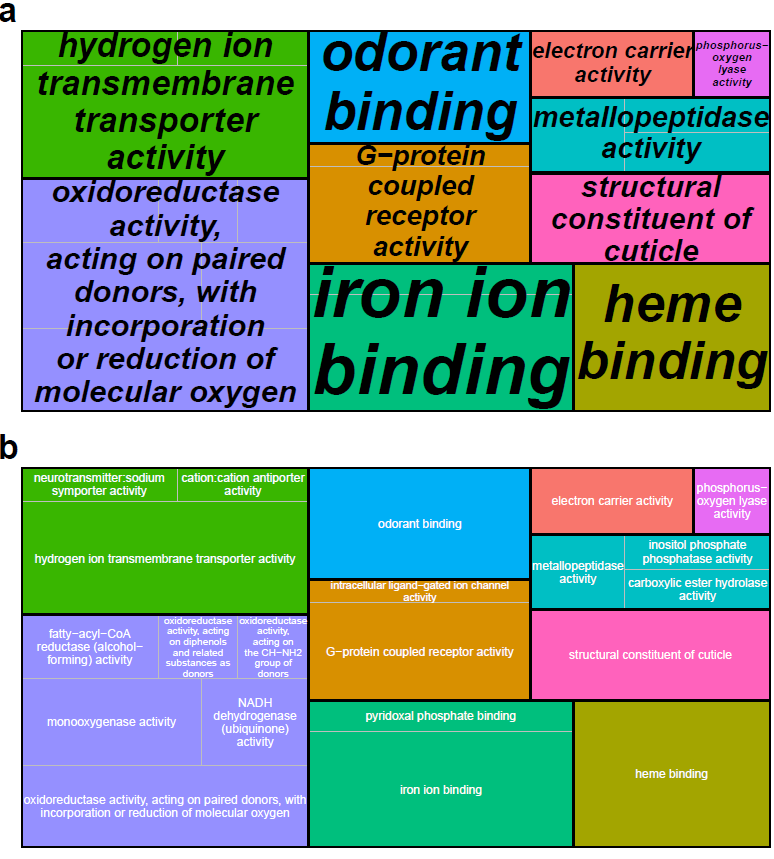


**Fig. S4:** Cellular Component treemap summarizing GO terms upregulated in adults. Enriched GO terms were reduced to the most specific terms in Blast2GO, and redundancy was further reduced using REVIGO. The size of each individual rectangle is relative to the absolute value of log_10_(FDR) for each GO term. a) Superclusters of related GO terms. b) All GO term cluster representatives.


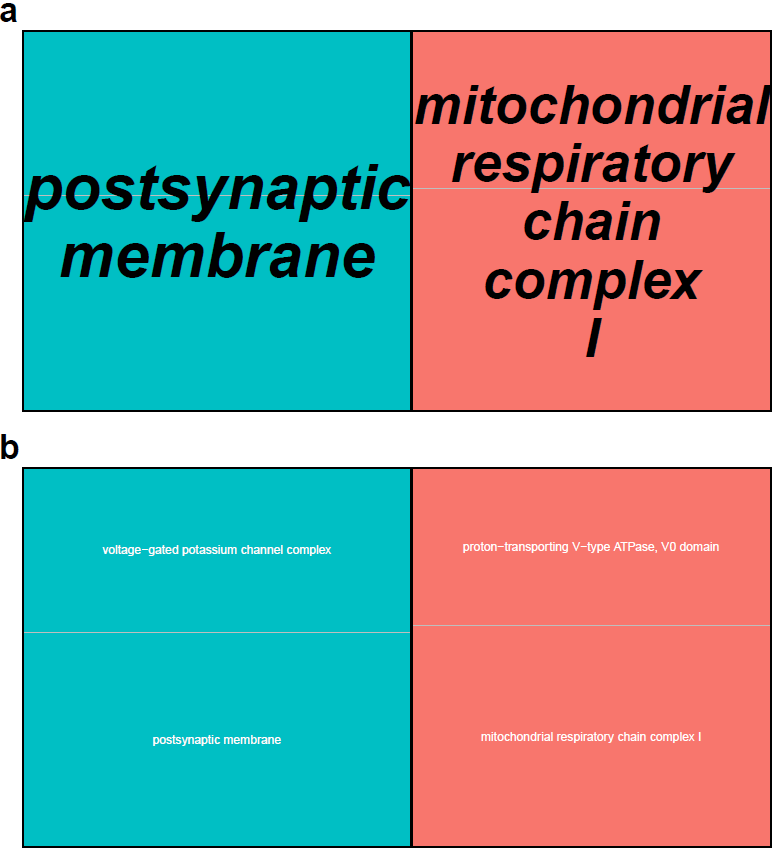


**Fig. S5:** Biological Process treemap summarizing GO terms upregulated in larvae. Enriched GO terms were reduced to the most specific terms in Blast2GO, and redundancy was further reduced using REVIGO. The size of each individual rectangle is relative to the absolute value of log_10_(FDR) for each GO term. a) Superclusters of related GO terms. b) All GO term cluster representatives.


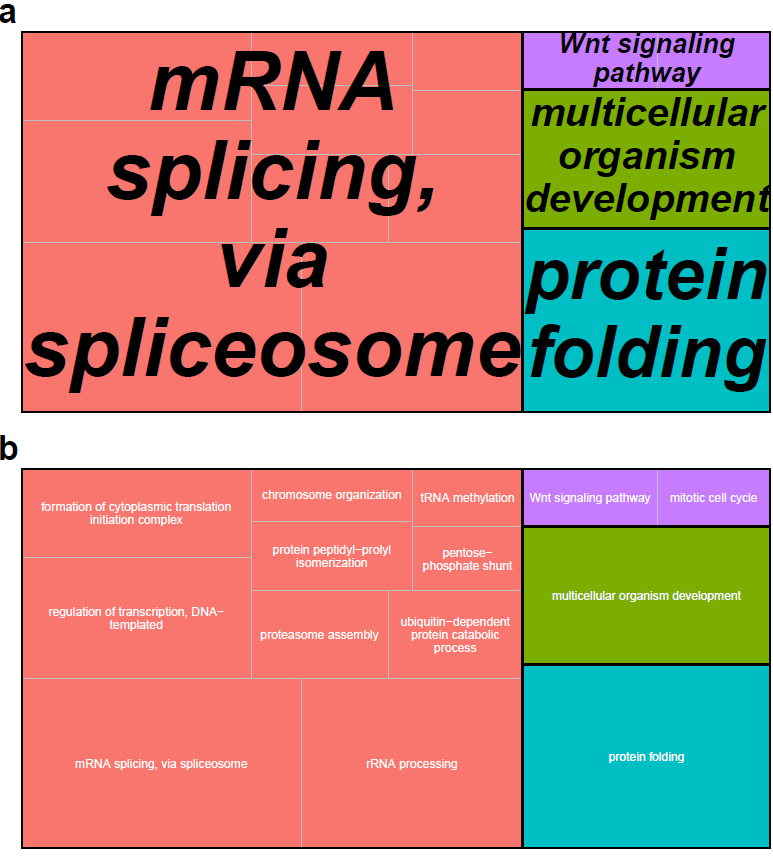


**Fig. S6:** Molecular Function treemap summarizing GO terms upregulated in larvae. Enriched GO terms were reduced to the most specific terms in Blast2GO, and redundancy was further reduced using REVIGO. The size of each individual rectangle is relative to the absolute value of log_10_(FDR) for each GO term. a) Superclusters of related GO terms. b) All GO term cluster representatives.


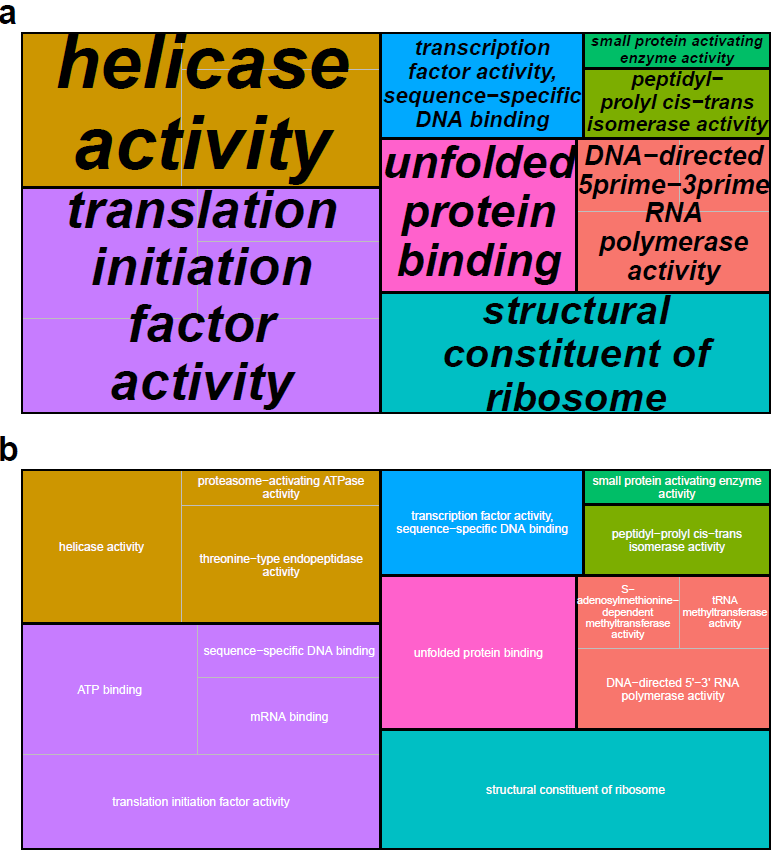


**Fig. S7:** Cellular Component treemap summarizing GO terms upregulated in larvae. Enriched GO terms were reduced to the most specific terms in Blast2GO, and redundancy was further reduced using REVIGO. The size of each individual rectangle is relative to the absolute value of log_10_(FDR) for each GO term. a) Superclusters of related GO terms. b) All GO term cluster representatives.


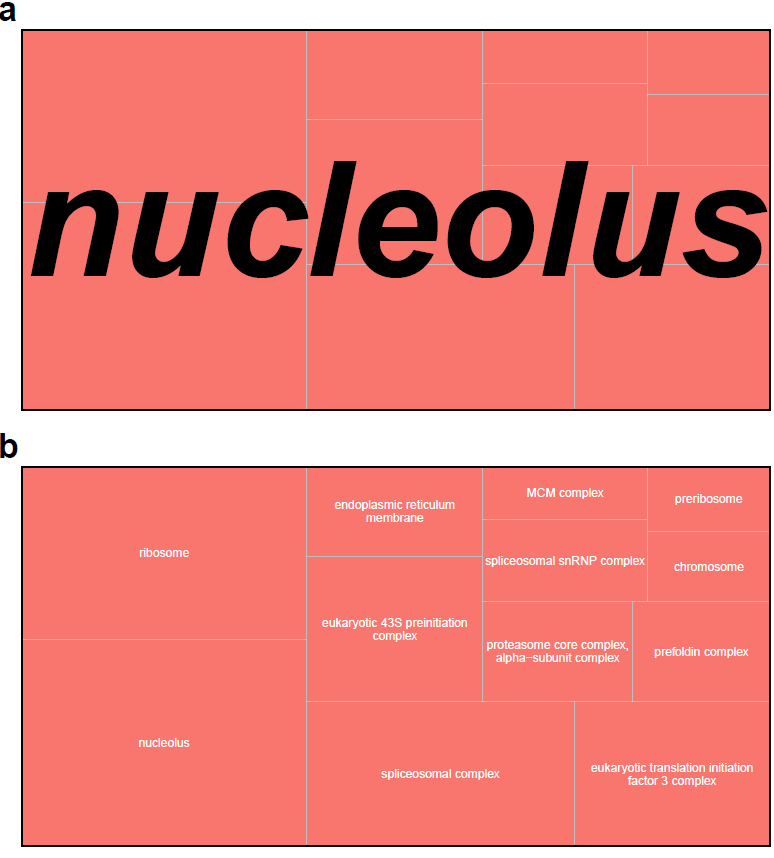


**Fig. S8:** Expression heatmap of additional differentially expressed genes putatively associated with vision. Counts were normalized by variance stabilizing transformation, with warmer colors indicating higher expression. Rows denote individual genes, and columns denote samples, both of which are clustered by gene expression. Family indicates the family from which the sample was derived, Sex indicates the sex of the sample, and Stage indicates the developmental stage of the sample.


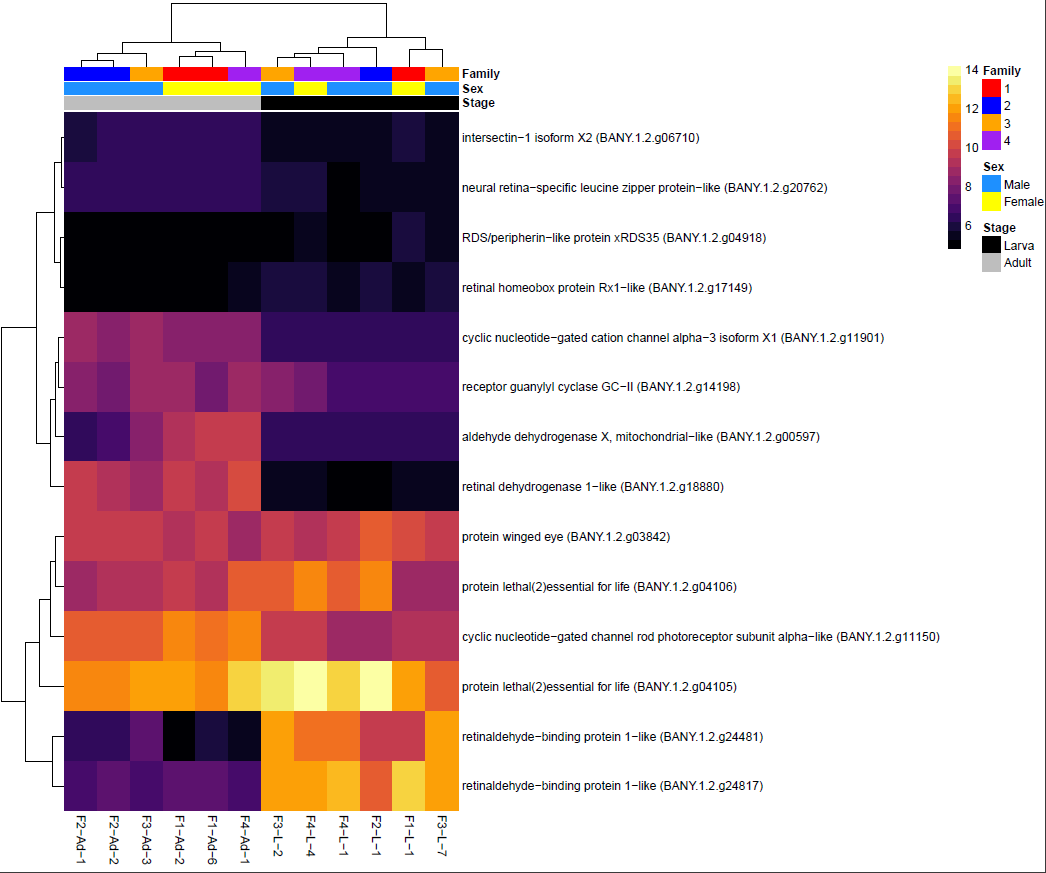


**Fig. S9:** Results of the larva sexing assay. The presence of a band at ~185 bp (red arrow) indicates amplification of a female-specific W-chromosome microsatellite, confirming that the sample is female. Male samples lack a band at this position. Samples to the left of the dashed line are adult positive controls (i.e., adults that were first sexed morphologically), while samples to the right of the dashed line are larvae. The first and last lanes are 100 bp ladders. NTC = no template control.


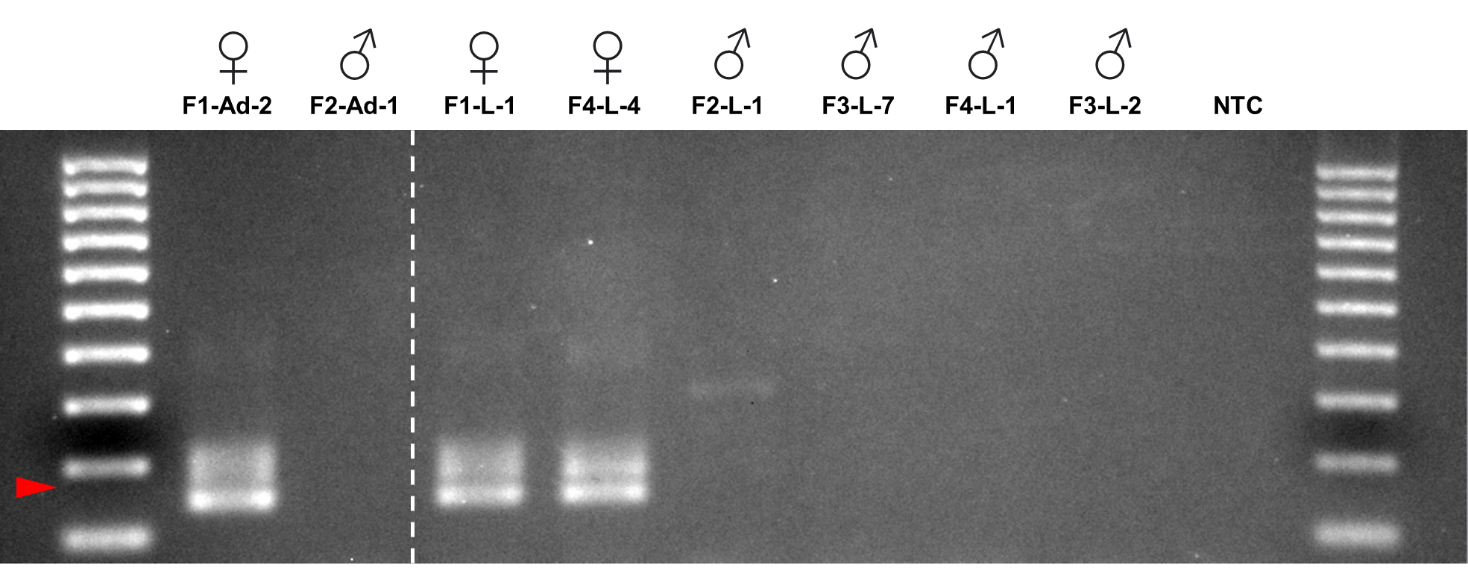

Supplement: Supplementary file 1 — Additional file 1: Supplemental Tables S1 and S25; captions for Supplemental Tables S2-S24; Supplemental Figures S1-S9. [file 12864_2021_7819_MOESM1_ESM.docx]
